# Supplementary material for: Model-based spatial-temporal mapping of opisthorchiasis in endemic countries of Southeast Asia
Source: eLife. 2021 Jan 12;10:e59755. doi: 10.7554/eLife.59755 (PMC7870142; doi:10.7554/eLife.59755)
Supplement: Figure 2—source data 2. [file elife-59755-fig2-data2.docx]

Figure 2-source data 2. $r_{t(K)}$ from the observed data and *p*-value of the preferential sampling test

| **Year** | $r_{t(K)}$ (*p*-value) | | | | | | | |
| --- | --- | --- | --- | --- | --- | --- | --- | --- |
|  | **K1** | **K2** | **K3** | **K4** | **K5** | **K6** | **K7** | **K8** |
| 1978 | -0.121 (0.50) | 0.075 (0.70) | 0.318 (0.15) | 0.370 (0.07) | 0.377 (0.06) | 0.366 (0.10) | 0.370 (0.09) | 0.372 (0.07) |
| 1981 | 0.322 (<0.001^*^) | 0.323 (<0.001^*^) | 0.294 (0.01^*^) | 0.276 (0.02^*^) | 0.263 (0.03^*^) | 0.268 (0.02^*^) | 0.271 (0.01^*^) | 0.268 (0.01^*^) |
| 1991 | -0.124 (0.37) | -0.126 (0.40) | -0.151 (0.35) | -0.200 (0.24) | -0.197 (0.26) | -0.206 (0.24) | -0.223 (0.20) | -0.224 (0.19) |
| 1995 | 0.044 (0.92) | 0.011 (0.97) | -0.029 (0.89) | -0.064 (0.82) | -0.076 (0.71) | -0.138 (0.58) | -0.161 (0.50) | -0.175 (0.44) |
| 1998 | -0.283 (0.30) | -0.419 (0.13) | -0.324 (0.18) | -0.124 (0.65) | -0.157 (0.57) | -0.115 (0.64) | -0.113 (0.69) | -0.127 (0.64) |
| 2000 | -0.091 (0.65) | -0.111 (0.66) | -0.122 (0.57) | -0.122 (0.53) | -0.102 (0.59) | -0.074 (0.69) | -0.054 (0.78) | -0.044 (0.89) |
| 2001 | -0.096 (0.44) | -0.040 (0.75) | -0.039 (0.78) | -0.083 (0.53) | -0.107 (0.43) | -0.119 (0.40) | -0.139 (0.36) | -0.156 (0.32) |
| 2004 | -0.400 (0.18) | -0.379 (0.18) | -0.375 (0.19) | -0.363 (0.18) | -0.363 (0.19) | -0.377 (0.19) | -0.403 (0.16) | -0.407 (0.12) |
| 2007 | -0.208 (0.50) | 0.235 (0.46) | 0.360 (0.32) | 0.310 (0.46) | 0.310 (0.44) | 0.310 (0.47) | 0.329 (0.41) | 0.290 (0.47) |
| 2008 | -0.406 (0.02^*^) | -0.370 (0.06) | -0.452 (<0.001^*^) | -0.475 (0.01^*^) | -0.471 (0.01^*^) | -0.404 (0.01^*^) | -0.330 (0.07) | -0.305 (0.09) |
| 2009 | 0.286 (0.01^*^) | 0.291 (0.01^*^) | 0.271 (0.04^*^) | 0.218 (0.11) | 0.194 (0.15) | 0.182 (0.20) | 0.177 (0.23) | 0.182 (0.22) |
| 2010 | -0.109 (0.55) | -0.298 (0.07) | -0.339 (0.03^*^) | -0.327 (0.03^*^) | -0.346 (0.01^*^) | -0.342 (0.02^*^) | -0.354 (<0.001^*^) | -0.359 (<0.001^*^) |
| 2011 | -0.051 (0.75) | 0.053 (0.80) | 0.081 (0.63) | 0.058 (0.71) | 0.052 (0.73) | 0.046 (0.77) | 0.057 (0.73) | 0.105 (0.51) |
| 2012 | 0.129 (0.53) | 0.142 (0.45) | 0.064 (0.69) | 0.043 (0.87) | 0.035 (0.90) | -0.012 (0.97) | 0.009 (0.95) | -0.023 (0.88) |
| 2013 | 0.133 (0.27) | 0.097 (0.48) | 0.080 (0.57) | 0.081 (0.56) | 0.061 (0.61) | 0.021 (0.90) | -0.006 (0.95) | -0.009 (0.95) |
| 2014 | 0.095 (0.38) | 0.181 (0.07) | 0.196 (0.07) | 0.204 (0.07) | 0.193 (0.08) | 0.188 (0.08) | 0.178 (0.11) | 0.166 (0.14) |
| 2015 | -0.435 (0.17) | -0.480 (0.17) | -0.553 (0.10) | -0.553 (0.08) | -0.553 (0.07) | -0.553 (0.06) | -0.553 (0.05) | -0.553 (0.04^*^) |
| 2016 | -0.622 (<0.001^*^) | -0.510 (<0.001^*^) | -0.511 (<0.001^*^) | -0.539 (<0.001^*^) | -0.536 (<0.01^*^) | -0.530 (<0.001^*^) | -0.516 (<0.001^*^) | -0.517 (<0.001^*^) |

Note: ^*^*p*<0.05, there is a significant preferential sampling.
