# Supplementary material for: Model-based spatial-temporal mapping of opisthorchiasis in endemic countries of Southeast Asia
Source: eLife. 2021 Jan 12;10:e59755. doi: 10.7554/eLife.59755 (PMC7870142; doi:10.7554/eLife.59755)
Supplement: Figure 2—figure supplement 1—source data 1. [file elife-59755-fig2-figsupp1-data1.docx]

**Quality assessment**

We did quality evaluation for each literature included in the final geostatistical modeling analysis, which is undertaken using a nine-point checklist. The items of quality evaluation are as follows:

Q1: provide specific inclusion and exclusion criteria.

Q2: provide basic characteristics of the investigated population (gender, age, etc.).

Q3: provide prevalence rate of the survey.

Q4: provide number of positive patients and number of examined people of the survey.

Q5: provide diagnostic method used in the survey.

Q6: provide survey type.

Q7: provide time of the survey.

Q8: describe or discuss the possible bias of the survey or how confounders are controlled.

Q9: the literature comes from Science Citation Index Expanded database.

Each item is scored 1 in case the publication meets) or 0 in contrary. The scores are summed up for all items and assigned to the publication as its quality score. The score for each literature is listed in Figure 2-figure supplement 1-source data 1.

**Figure 2-figure supplement 1-source data 1.** **Quality assessment for eligible literatures**

| **Literature (first author & year of publication)** | **Q1** | **Q2** | **Q3** | **Q4** | **Q5** | **Q6** | **Q7** | **Q8** | **Q9** | **Total score** |
| --- | --- | --- | --- | --- | --- | --- | --- | --- | --- | --- |
| ***Viyanant, et al.***,***1983*** | 1 | 0 | 1 | 1 | 1 | 1 | 1 | 1 | 0 | 7 |
| ***Waikagul, et al.***,***2002*** | 1 | 0 | 1 | 1 | 1 | 1 | 1 | 0 | 0 | 6 |
| ***Assavapongpaiboon, et al.***,***2018*** | 1 | 1 | 1 | 0 | 1 | 1 | 1 | 0 | 1 | 7 |
| ***Boonjaraspinyo, et al.***,***2013*** | 1 | 1 | 1 | 1 | 1 | 1 | 1 | 0 | 1 | 8 |
| ***Suwannahitatorn, et al.***,***2013*** | 1 | 1 | 1 | 1 | 1 | 1 | 1 | 1 | 1 | 9 |
| ***Prakobwong, et al.***,***2017*** | 1 | 1 | 1 | 1 | 1 | 1 | 1 | 0 | 0 | 7 |
| ***Traub, et al.***,***2009*** | 1 | 0 | 1 | 1 | 1 | 1 | 1 | 1 | 1 | 8 |
| ***Sornmani, et al.***,***1984*** | 0 | 0 | 1 | 1 | 0 | 1 | 1 | 0 | 0 | 4 |
| ***Chai and Hongvanthong***,***1998*** | 1 | 1 | 1 | 1 | 1 | 1 | 1 | 1 | 0 | 8 |
| ***Jadsri and Noojoy***,***1999*** | 1 | 1 | 1 | 1 | 0 | 1 | 1 | 0 | 0 | 6 |
| ***Sohn, et al.***,***2011*** | 1 | 0 | 1 | 1 | 1 | 1 | 1 | 1 | 1 | 8 |
| ***Worasith, et al.***,***2015*** | 1 | 1 | 1 | 1 | 1 | 1 | 1 | 1 | 1 | 9 |
| ***Kobayashi, et al.***,***2000*** | 0 | 1 | 1 | 1 | 1 | 1 | 1 | 0 | 0 | 6 |
| ***Vannachone, et al.***,***1998*** | 0 | 1 | 1 | 1 | 1 | 1 | 1 | 0 | 0 | 6 |
| ***Sornmani, et al.***,***1981*** | 1 | 1 | 1 | 1 | 0 | 1 | 0 | 0 | 0 | 5 |
| ***Kaewpitoon, et al.***,***2016*** | 1 | 1 | 1 | 1 | 1 | 1 | 1 | 0 | 0 | 7 |
| ***Bukkhunthod, et al.***,***2020*** | 1 | 1 | 1 | 1 | 0 | 1 | 1 | 0 | 0 | 6 |
| ***Wang, et al.***,***2017*** | 0 | 0 | 1 | 0 | 0 | 1 | 1 | 0 | 1 | 4 |
| ***Htun, et al.***,***2018*** | 1 | 1 | 1 | 1 | 1 | 1 | 1 | 1 | 1 | 9 |
| ***Van CD, et al.***,***2017*** | 1 | 1 | 1 | 1 | 0 | 1 | 0 | 0 | 1 | 6 |
| ***Erlanger, et al.***,***2008*** | 1 | 1 | 1 | 1 | 0 | 1 | 0 | 0 | 1 | 6 |
| ***Viravan, et al.***,***1986*** | 0 | 1 | 1 | 1 | 0 | 1 | 0 | 0 | 0 | 4 |
| ***Doanh and Nawa***,***2016*** | 0 | 0 | 1 | 0 | 0 | 1 | 0 | 0 | 1 | 3 |
| ***Wongsawad, et al.***,***2012*** | 1 | 1 | 1 | 1 | 1 | 1 | 0 | 0 | 1 | 7 |
| ***Kaewpitoon, et al.***,***2012*** | 1 | 1 | 1 | 1 | 1 | 1 | 1 | 0 | 1 | 8 |
| ***Worasith, et al.***,***2019*** | 1 | 1 | 1 | 1 | 1 | 1 | 1 | 0 | 1 | 8 |
| ***Vonghachack, et al.***,***2017*** | 1 | 1 | 1 | 1 | 1 | 1 | 1 | 1 | 1 | 9 |
| ***Charoensuk, et al.***,***2019*** | 1 | 0 | 1 | 1 | 1 | 1 | 1 | 0 | 1 | 7 |
| ***Sato, et al.***,***2010*** | 0 | 0 | 1 | 1 | 1 | 1 | 1 | 1 | 1 | 7 |
| ***Yajima, et al.***,***2009*** | 1 | 1 | 1 | 1 | 1 | 1 | 1 | 0 | 1 | 8 |
| ***Haswell-Elkins, et al.***,***1994*** | 1 | 1 | 1 | 1 | 1 | 1 | 1 | 1 | 0 | 8 |
| ***Laoraksawong, et al.***,***2018*** | 1 | 1 | 1 | 1 | 1 | 1 | 1 | 0 | 1 | 8 |
| ***Hung, et al.***,***2015*** | 1 | 1 | 1 | 1 | 1 | 1 | 1 | 1 | 1 | 9 |
| ***Giboda, et al.***,***1991*** | 1 | 1 | 1 | 1 | 1 | 0 | 1 | 0 | 0 | 6 |
| ***Laymanivong, et al.***,***2014*** | 1 | 0 | 1 | 1 | 1 | 1 | 1 | 1 | 1 | 8 |
| ***Kaewpitoon, et al.***,***2018*** | 1 | 1 | 1 | 1 | 1 | 1 | 1 | 0 | 0 | 7 |
| ***Khieu, et al.***,***2013*** | 1 | 1 | 1 | 1 | 1 | 1 | 1 | 1 | 1 | 9 |
| ***Haswell-Elkins, et al.***,***1991*** | 1 | 1 | 1 | 1 | 1 | 1 | 1 | 0 | 0 | 7 |
| ***Sohn, et al.***,***2011*** | 1 | 0 | 1 | 1 | 1 | 1 | 1 | 1 | 1 | 8 |
| ***Soukhathammavong, et al.***,***2011*** | 1 | 0 | 1 | 1 | 0 | 1 | 1 | 1 | 1 | 7 |
| ***Salao, et al.***,***2020*** | 1 | 0 | 1 | 1 | 1 | 0 | 0 | 0 | 1 | 5 |
| ***Wang, et al.***,***2013*** | 1 | 1 | 1 | 0 | 0 | 1 | 1 | 0 | 1 | 6 |
| ***Doi, et al.***,***2017*** | 1 | 1 | 1 | 1 | 1 | 1 | 1 | 0 | 0 | 7 |
| ***Sithithaworn, et al.***,***2006*** | 1 | 1 | 1 | 1 | 1 | 1 | 1 | 0 | 0 | 7 |
| ***Sayasone, et al.***,***2007*** | 1 | 1 | 1 | 0 | 1 | 1 | 1 | 1 | 1 | 8 |
| ***Prakobwong, et al.***,***2017*** | 1 | 1 | 1 | 1 | 1 | 1 | 1 | 0 | 1 | 8 |
| ***Suwannatrai, et al.***,***2018*** | 0 | 0 | 0 | 0 | 0 | 1 | 1 | 0 | 1 | 3 |
| ***Sithithaworn, et al.***,***2003*** | 1 | 1 | 1 | 1 | 1 | 1 | 1 | 1 | 1 | 9 |
| ***Vonghachack, et al.***,***2015*** | 1 | 1 | 1 | 1 | 1 | 1 | 1 | 1 | 1 | 9 |
| ***Laoprom, et al.***,***2016*** | 1 | 1 | 1 | 1 | 1 | 1 | 1 | 1 | 1 | 9 |
| ***Wangboon, et al.***,***2019*** | 1 | 1 | 1 | 1 | 1 | 1 | 1 | 0 | 1 | 8 |
| ***Miyamoto, et al.***,***2014*** | 1 | 1 | 1 | 1 | 1 | 1 | 1 | 1 | 1 | 9 |
| ***Saowakontha, et al.***,***1993*** | 1 | 1 | 1 | 1 | 1 | 1 | 1 | 0 | 0 | 7 |
| ***Laymanivong, et al.***,***2016*** | 1 | 1 | 1 | 1 | 1 | 1 | 0 | 0 | 1 | 7 |
| ***Aung, et al.***,***2017*** | 1 | 1 | 1 | 1 | 1 | 1 | 1 | 1 | 1 | 9 |
| ***Saenna, et al.***,***2017*** | 1 | 1 | 1 | 1 | 1 | 1 | 0 | 0 | 1 | 7 |
| ***Watthanakulpanich, et al.***,***2010*** | 1 | 0 | 1 | 1 | 1 | 1 | 0 | 1 | 1 | 7 |
| ***Sayasone, et al.***,***2011*** | 1 | 1 | 1 | 1 | 1 | 1 | 1 | 1 | 1 | 9 |
| ***Phongluxa, et al.***,***2013*** | 1 | 1 | 1 | 1 | 1 | 1 | 1 | 1 | 1 | 9 |
| ***Wiwanitkit, et al.***,***2002*** | 1 | 0 | 1 | 1 | 0 | 1 | 0 | 0 | 0 | 4 |
| ***Wijit, et al.***,***2013*** | 1 | 0 | 1 | 1 | 1 | 1 | 0 | 1 | 1 | 7 |
| ***Chai, et al.***,***2009*** | 1 | 1 | 1 | 1 | 1 | 1 | 1 | 1 | 1 | 9 |
| ***Bless, et al.***,***2015*** | 1 | 1 | 1 | 1 | 1 | 1 | 1 | 1 | 1 | 9 |
| ***Chai, et al.***,***2007*** | 1 | 1 | 1 | 1 | 1 | 1 | 1 | 0 | 0 | 7 |
| ***Yong, et al.***,***2012*** | 1 | 1 | 1 | 1 | 1 | 1 | 1 | 1 | 1 | 9 |
| ***De NV and Le TH***,***2011*** | 1 | 1 | 1 | 1 | 1 | 1 | 1 | 1 | 1 | 9 |
| ***Chai, et al.***,***2013*** | 1 | 0 | 1 | 1 | 1 | 1 | 1 | 1 | 1 | 8 |
| ***Watwiengkam, et al.***,***2013*** | 1 | 0 | 1 | 1 | 1 | 1 | 0 | 1 | 1 | 7 |
| ***Duenngai, et al.***,***2008*** | 0 | 0 | 1 | 1 | 1 | 1 | 0 | 1 | 1 | 6 |
| ***Rangsin, et al.***,***2009*** | 1 | 1 | 1 | 1 | 1 | 1 | 1 | 1 | 1 | 9 |
| ***Sornmani, et al.***,***1984*** | 1 | 0 | 1 | 1 | 1 | 1 | 1 | 0 | 0 | 6 |
| ***Saengsawang, et al.***,***2013*** | 1 | 1 | 1 | 1 | 1 | 1 | 1 | 1 | 1 | 9 |
| ***Sinuon, et al.***,***2003*** | 1 | 1 | 1 | 1 | 1 | 1 | 0 | 0 | 0 | 6 |
| ***Chai, et al.***,***2015*** | 1 | 0 | 1 | 1 | 1 | 1 | 1 | 0 | 1 | 7 |
| ***Warunee, et al.***,***2007*** | 1 | 1 | 1 | 1 | 1 | 1 | 1 | 1 | 1 | 9 |
| ***Kasuya, et al.***,***1989*** | 1 | 1 | 1 | 1 | 0 | 1 | 0 | 0 | 0 | 5 |
| ***Ribas, et al.***,***2017*** | 1 | 1 | 1 | 1 | 1 | 1 | 0 | 0 | 1 | 7 |
| ***Piangjai, et al.***,***2003*** | 1 | 1 | 1 | 1 | 1 | 1 | 1 | 1 | 1 | 9 |
| ***Wongba, et al.***,***2011*** | 1 | 0 | 1 | 1 | 1 | 1 | 0 | 1 | 1 | 7 |
| ***Strandgaard, et al.***,***2008*** | 1 | 1 | 1 | 0 | 0 | 1 | 1 | 0 | 0 | 5 |
| ***Schurer, et al.***,***2019*** | 0 | 1 | 1 | 1 | 0 | 1 | 0 | 0 | 1 | 5 |
| ***Sohn, et al.***,***2019*** | 0 | 0 | 1 | 1 | 1 | 0 | 1 | 0 | 1 | 5 |
| ***Brockelman, et al.***,***1987*** | 1 | 1 | 1 | 1 | 1 | 1 | 1 | 0 | 0 | 7 |
| ***Chai, et al.***,***2005*** | 1 | 1 | 1 | 1 | 1 | 1 | 1 | 1 | 1 | 9 |
| ***León, et al.***,***2018*** | 1 | 0 | 1 | 0 | 0 | 1 | 1 | 1 | 1 | 6 |
| ***Buathong, et al.***,***2017*** | 1 | 0 | 1 | 1 | 1 | 1 | 1 | 1 | 1 | 8 |
| ***Feldmeier, et al.***,***2016*** | 1 | 1 | 1 | 1 | 1 | 1 | 0 | 1 | 0 | 7 |
| ***Upatham, et al.***,***1982*** | 1 | 1 | 1 | 0 | 1 | 1 | 1 | 0 | 1 | 7 |
| ***Araki, et al.***,***2018*** | 1 | 1 | 1 | 1 | 1 | 1 | 1 | 0 | 0 | 7 |
| ***Sayasone, et al.***,***2015*** | 1 | 1 | 1 | 1 | 1 | 1 | 1 | 1 | 1 | 9 |
| ***Kaewpitoon, et al.***,***2016*** | 1 | 1 | 1 | 1 | 1 | 1 | 1 | 0 | 0 | 7 |
| ***Uengarporn, et al.***,***2016*** | 1 | 1 | 1 | 1 | 1 | 1 | 1 | 0 | 0 | 7 |
| ***Radomyos, et al.***,***1998*** | 1 | 0 | 1 | 1 | 1 | 1 | 0 | 1 | 0 | 6 |
| ***Kaewpitoon, et al.***,***2016*** | 1 | 1 | 1 | 1 | 1 | 1 | 1 | 0 | 0 | 7 |
| ***Nithikathkul, et al.***,***2009*** | 1 | 1 | 1 | 1 | 1 | 1 | 1 | 0 | 1 | 8 |
| ***Pungpak, et al.***,***1994*** | 1 | 1 | 1 | 1 | 0 | 1 | 1 | 0 | 0 | 6 |
| ***Dao, et al.***,***2016*** | 1 | 1 | 1 | 1 | 1 | 1 | 1 | 1 | 1 | 9 |
| ***Srivatanakul, et al.***,***1991*** | 1 | 1 | 1 | 1 | 1 | 1 | 1 | 0 | 0 | 7 |
| ***Kaewpitoon, et al.***,***2016*** | 1 | 1 | 1 | 1 | 1 | 1 | 1 | 0 | 0 | 7 |
| ***Chaisiri, et al.***,***2018*** | 1 | 1 | 1 | 1 | 1 | 1 | 1 | 1 | 1 | 9 |
| ***Sato, et al.***,***2015*** | 1 | 1 | 1 | 1 | 1 | 1 | 1 | 0 | 1 | 8 |
| ***Lovis, et al.***,***2009*** | 1 | 0 | 1 | 1 | 1 | 1 | 1 | 0 | 1 | 7 |
| ***Bunnag, et al.***,***1980*** | 1 | 1 | 1 | 1 | 1 | 1 | 1 | 0 | 0 | 7 |
| ***Pumidonming, et al.***,***2018*** | 1 | 1 | 1 | 1 | 1 | 1 | 1 | 1 | 1 | 9 |
| ***Yoshida, et al.***,***2019*** | 1 | 1 | 1 | 1 | 1 | 1 | 1 | 1 | 1 | 9 |
| ***Nakamura***,***2017*** | 1 | 0 | 1 | 1 | 1 | 1 | 1 | 0 | 0 | 6 |
| ***Niamnuy, et al.***,***2016*** | 1 | 1 | 1 | 1 | 1 | 1 | 0 | 0 | 1 | 7 |
| ***Chuangchaiya, et al.***,***2019*** | 1 | 1 | 1 | 1 | 1 | 1 | 1 | 1 | 1 | 9 |
| ***Songserm, et al.***,***2012*** | 1 | 1 | 1 | 1 | 1 | 1 | 1 | 0 | 1 | 8 |
| ***Saiyachak, et al.***,***2016*** | 1 | 1 | 1 | 1 | 1 | 1 | 1 | 1 | 0 | 8 |
| ***Maleewong, et al.***,***1992*** | 1 | 1 | 1 | 1 | 1 | 1 | 1 | 0 | 0 | 7 |
| ***Chaiputcha, et al.***,***2015*** | 1 | 1 | 1 | 1 | 1 | 1 | 1 | 1 | 0 | 8 |
| ***Thaewnongiew, et al.***,***2014*** | 1 | 1 | 0 | 0 | 1 | 1 | 1 | 0 | 1 | 6 |
| ***Khieu, et al.***,***2014*** | 1 | 1 | 1 | 1 | 1 | 1 | 1 | 1 | 1 | 9 |
| ***Sohn, et al.***,***2014*** | 1 | 1 | 1 | 1 | 1 | 1 | 1 | 1 | 1 | 9 |
| ***Eom, et al.***,***2014*** | 1 | 1 | 1 | 1 | 1 | 1 | 1 | 1 | 1 | 9 |
| ***Ruankham, et al.***,***2014*** | 1 | 0 | 1 | 1 | 1 | 1 | 1 | 0 | 0 | 6 |
| ***Yong, et al.***,***2014*** | 1 | 1 | 1 | 1 | 1 | 1 | 1 | 1 | 1 | 9 |
| ***Kobayashi, et al.***,***1999*** | 1 | 1 | 1 | 1 | 1 | 1 | 1 | 1 | 1 | 9 |
| ***Rim, et al.***,***2003*** | 1 | 0 | 1 | 1 | 1 | 1 | 1 | 1 | 1 | 8 |
| ***Kobayashi, et al.***,***1996*** | 1 | 1 | 1 | 1 | 0 | 1 | 0 | 0 | 0 | 5 |
| ***Joob and Wiwanitkit***,***2017*** | 0 | 0 | 1 | 0 | 0 | 0 | 0 | 0 | 0 | 1 |
| ***Sukontason, et al.***,***2001*** | 1 | 0 | 1 | 1 | 1 | 1 | 1 | 0 | 0 | 6 |
| ***Sriamporn, et al.***,***2004*** | 1 | 1 | 1 | 1 | 1 | 1 | 1 | 1 | 1 | 9 |
| ***Sohn, et al.***,***2012*** | 1 | 1 | 1 | 1 | 1 | 1 | 1 | 1 | 1 | 9 |
| ***Kaewpitoon, et al.***,***2012*** | 1 | 1 | 1 | 1 | 1 | 1 | 1 | 0 | 1 | 8 |
| ***Saengsawang, et al.***,***2012*** | 1 | 1 | 1 | 1 | 1 | 1 | 1 | 1 | 1 | 9 |
| ***Nontasut, et al.***,***2005*** | 1 | 0 | 1 | 1 | 1 | 1 | 1 | 0 | 0 | 6 |
| ***Chai, et al.***,***2010*** | 1 | 0 | 1 | 1 | 1 | 1 | 1 | 1 | 1 | 8 |
| ***Dang, et al.***,***2008*** | 1 | 1 | 1 | 1 | 1 | 1 | 1 | 0 | 1 | 8 |
| ***Kaewpitoon, et al.***,***2016*** | 1 | 0 | 1 | 1 | 1 | 1 | 1 | 0 | 0 | 6 |
| ***Kaewpitoon, et al.***,***2016*** | 1 | 1 | 1 | 1 | 1 | 1 | 1 | 0 | 0 | 7 |
| ***Yeoh, et al.***,***2015*** | 1 | 1 | 1 | 1 | 1 | 1 | 1 | 1 | 0 | 8 |
| ***Upatham, et al.***,***1984*** | 1 | 1 | 1 | 1 | 1 | 1 | 1 | 0 | 0 | 7 |
| ***Rujirakul, et al.***,***2015*** | 1 | 0 | 1 | 1 | 0 | 1 | 1 | 0 | 0 | 5 |
| ***Yoon, et al.***,***2014*** | 1 | 1 | 1 | 1 | 1 | 1 | 1 | 0 | 1 | 8 |
| ***Tomokawa, et al.***,***2012*** | 1 | 1 | 1 | 1 | 1 | 1 | 1 | 0 | 1 | 8 |
| ***Akai, et al.***,***1995*** | 1 | 0 | 1 | 0 | 0 | 1 | 0 | 0 | 0 | 3 |
| ***Phimpraphai, et al.***,***2017*** | 1 | 1 | 1 | 0 | 1 | 1 | 0 | 0 | 1 | 6 |
| ***Forrer, et al.***,***2012*** | 0 | 0 | 1 | 0 | 0 | 1 | 1 | 0 | 1 | 4 |
| ***Lee, et al.***,***2002*** | 1 | 1 | 1 | 1 | 1 | 1 | 1 | 0 | 0 | 7 |
| ***Aye, et al.***,***2015*** | 1 | 1 | 1 | 1 | 1 | 1 | 1 | 1 | 1 | 9 |
| ***Kaewpitoon, et al.***,***2015*** | 1 | 1 | 1 | 1 | 1 | 1 | 1 | 0 | 0 | 7 |
| ***Sithithaworn, et al.***,***2012*** | 0 | 0 | 1 | 0 | 0 | 1 | 0 | 0 | 1 | 3 |
| ***Tesana, et al.***,***2007*** | 1 | 0 | 1 | 1 | 1 | 1 | 0 | 0 | 0 | 5 |
| ***Sripa, et al.***,***2017*** | 0 | 0 | 1 | 0 | 0 | 1 | 0 | 0 | 1 | 3 |
| ***Tungtrongchitr, et al.***,***2007*** | 1 | 0 | 1 | 1 | 1 | 1 | 0 | 1 | 0 | 6 |
| ***Waree, et al.***,***2001*** | 1 | 1 | 1 | 1 | 1 | 1 | 1 | 0 | 0 | 7 |
| ***Schär, et al.***,***2014*** | 1 | 1 | 1 | 1 | 1 | 1 | 1 | 1 | 1 | 9 |
| ***Kaewpitoon, et al.***,***2019*** | 0 | 1 | 1 | 1 | 1 | 1 | 1 | 1 | 0 | 7 |
| ***Echaubard, et al.***,***2016*** | 0 | 0 | 1 | 0 | 0 | 1 | 1 | 0 | 1 | 4 |
| ***Srivatanakul, et al.***,***1991*** | 1 | 1 | 1 | 1 | 1 | 1 | 0 | 1 | 0 | 7 |
| ***Khampitak, et al.***,***2006*** | 1 | 1 | 1 | 1 | 1 | 1 | 0 | 0 | 0 | 6 |
| ***Sripa, et al.***,***2015*** | 0 | 0 | 1 | 0 | 0 | 1 | 1 | 0 | 1 | 4 |
| ***Vonghachack, et al.***,***2017*** | 1 | 1 | 1 | 1 | 1 | 1 | 1 | 1 | 1 | 9 |
| ***Kim, et al.***,***2018*** | 1 | 1 | 1 | 1 | 1 | 1 | 1 | 1 | 1 | 9 |
| ***Pungpak, et al.***,***1989*** | 1 | 1 | 1 | 1 | 1 | 1 | 1 | 0 | 0 | 7 |
| ***Mairiang, et al.***,***2012*** | 1 | 1 | 1 | 1 | 1 | 1 | 1 | 0 | 1 | 8 |
| ***Sungkasubun, et al.***,***2016*** | 1 | 1 | 1 | 1 | 0 | 1 | 1 | 1 | 1 | 8 |
| ***Ong, et al.***,***2016*** | 1 | 1 | 1 | 1 | 0 | 1 | 1 | 0 | 1 | 7 |
| ***Chuong and Tuan***,***1994*** (in Vietnamese) | 0 | 0 | 1 | 1 | 0 | 1 | 0 | 0 | 0 | 3 |
| ***Chuong, et al.***,***2000*** (in Vietnamese) | 0 | 0 | 1 | 1 | 0 | 1 | 0 | 0 | 0 | 3 |
| ***Chuong, et al.***,***1997*** (in Vietnamese) | 0 | 0 | 1 | 1 | 0 | 1 | 0 | 0 | 0 | 3 |
| ***Chuong, et al.***,***2014*** (in Vietnamese) | 0 | 0 | 1 | 1 | 0 | 1 | 0 | 0 | 0 | 3 |
| ***Chuong and Tuan***,***2011*** (in Vietnamese) | 0 | 0 | 1 | 0 | 0 | 1 | 0 | 0 | 0 | 2 |
| ***De, et al.***,***1997*** (in Vietnamese) | 0 | 0 | 1 | 1 | 0 | 1 | 0 | 0 | 0 | 3 |
| ***De, et al.***,***1996*** (in Vietnamese) | 0 | 0 | 1 | 1 | 0 | 1 | 0 | 0 | 0 | 3 |
| ***De, et al.***,***1998*** (in Vietnamese) | 0 | 0 | 1 | 1 | 0 | 1 | 0 | 0 | 0 | 3 |

**References**

Akai PS, Pungpak S, Chaicumpa W, Kitikoon V, Ruangkunaporn Y, Bunnag D, Befus AD. 1995. Serum antibody responses in opisthorchiasis. *International Journal for Parasitology* **25**:971-973. doi: 10.1016/0020-7519(94)00212-7

Araki H, Ong K, Lorphachan L, Soundala P, Iwagami M, Shibanuma A, Hongvanthong B, Brey PT, Kano S, Jimba M. 2018. Mothers' *Opisthorchis viverrini* infection status and raw fish dish consumption in Lao People's Democratic Republic: determinants of child infection status. *Tropical Medicine and Health* **46**:29. doi:10.1186/s41182-018-0112-y

Assavapongpaiboon B, Bunkasem U, Sanprasert V, Nuchprayoon S. 2018. A Cross-Sectional Study on Intestinal Parasitic Infections in Children in Suburban Public Primary Schools, Saraburi, the Central Region of Thailand. *The American journal of tropical medicine and hygiene* **98**:763-767. doi:10.4269/ajtmh.17-0240

Aung WPP, Htoon TT, Tin HH, Thinn KK, Sanpool O, Jongthawin J, Sadaow L, Phosuk I, Rodpai R, Intapan PM, Maleewong W. 2017. First report and molecular identification of *Opisthorchis viverrini* infection in human communities from Lower Myanmar. *PLOS ONE* **12**:e177130. doi:10.1371/journal.pone.0177130

Aye SP, Rajpho V, Phongluxa K, Vonghachack Y, Hattendorf J, Hongvanthong B, Rasaphon O, Sripa B, Akkhavong K, Hatz C, Odermatt P. 2015. Subtle to severe hepatobiliary morbidity in *Opisthorchis viverrini* endemic settings in southern Laos. *Acta Tropica* **141**:303-309. doi:10.1016/j.actatropica.2014.09.014

Bless PJ, Schar F, Khieu V, Kramme S, Muth S, Marti H, Odermatt P. 2015. High prevalence of large trematode eggs in schoolchildren in Cambodia. *Acta Tropica* **141**:295-302. doi:10.1016/j.actatropica.2014.09.007

Boonjaraspinyo S, Boonmars T, Kaewsamut B, Ekobol N, Laummaunwai P, Aukkanimart R, Wonkchalee N, Juasook A, Sriraj P. 2013. A Cross-Sectional Study on Intestinal Parasitic Infections in Rural Communities, Northeast Thailand. *The Korean Journal of Parasitology* **51**:727-734. doi:10.3347/kjp.2013.51.6.727

Brockelman WY, Upatham ES, Viyanant V, Hirunraks A. 1987. Measurement of incidence of the human liver fluke, *Opisthorchis viverrini*, in northeast Thailand. *Transactions of the Royal Society of Tropical Medicine and Hygiene* **81**:327. doi: 10.1016/0035-9203(87)90255-0

Buathong S, Leelayoova S, Mungthin M, Ruang-Areerate T, Naaglor T, Suwannahitatorn P, Piyaraj P, Taamasri P, Tan-Ariya P. 2017. Molecular discrimination of Opisthorchis-like eggs from residents in a rural community of central Thailand. *PLOS Neglected Tropical Diseases* **11**:e6030. doi:10.1371/journal.pntd.0006030

Bukkhunthod P, Meererksom T, Pechdee P, Ponphimai S, Khiaowichit J, Kaewpitoon N, Thueng-In K, Leng M, Namhong T, Taweepakdeechot A, Yardcharoen N, Srithongklang W, Wakhuwathapong P, Keeratibharat N, Kaewpitoon SJ. 2020. Animation as Supplementary Learning Material About Carcinogenic Liver Fluke in Classes for Primary Schoolchildren. *Journal of cancer education : the official journal of the American Association for Cancer Education* **35**:14-21. doi:10.1007/s13187-018-1434-5

Bunnag T, Sornmani S, Impand P, Harinasuta C. 1980. Potential health hazards of the water resources development: a health survey in the Phitsanulok Irrigation Project, Nan River Basin, Northern Thailand. *The Southeast Asian Journal of Tropical Medicine and Public Health* **11**:559-565. PMID: 7221699

Chai JY, Han ET, Guk SM, Shin EH, Sohn WM, Yong TS, Eom KS, Lee KH, Jeong HG, Ryang YS, Hoang EH, Phommasack B, Insisiengmay B, Lee SH, Rim HJ. 2007. High prevalence of liver and intestinal fluke infections among residents of Savannakhet Province in Laos. *The Korean Journal of Parasitology* **45**:213-218. doi:10.3347/kjp.2007.45.3.213

Chai JY, Han ET, Shin EH, Sohn WM, Yong TS, Eom KS, Min DY, Um JY, Park MS, Hoang EH, Phommasack B, Insisiengmay B, Lee SH, Rim HJ. 2009. High prevalence of *Haplorchis taichui*, Phaneropsolus molenkampi, and other helminth infections among people in Khammouane province, Lao PDR. *The Korean Journal of Parasitology* **47**:243-247. doi:10.3347/kjp.2009.47.3.243

Chai JY, Hongvanthong B. 1998. A small-scale survey of intestinal helminthic infections among the residents near Pakse, Laos. *The Korean Journal of Parasitology* **36**:55-58. doi:10.3347/kjp.1998.36.1.55

Chai JY, Park JH, Han ET, Guk SM, Shin EH, Lin A, Kim JL, Sohn WM, Yong TS, Eom KS, Min DY, Hwang EH, Phommmasack B, Insisiengmay B, Rim HJ. 2005. Mixed infections with *Opisthorchis viverrini* and intestinal flukes in residents of Vientiane Municipality and Saravane Province in Laos. *Journal of Helminthology* **79**:283-289. doi:10.1079 / joh2005302

Chai JY, Sohn WM, Jung BK, Yong TS, Eom KS, Min DY, Insisiengmay B, Insisiengmay S, Phommasack B, Rim HJ. 2015. Intestinal Helminths Recovered from Humans in Xieng Khouang Province, Lao PDR with a Particular Note on *Haplorchis pumilio* Infection. *The Korean Journal of Parasitology* **53**:439-445. doi:10.3347/kjp.2015.53.4.439

Chai JY, Yong TS, Eom KS, Min DY, Jeon HK, Kim TY, Jung BK, Sisabath L, Insisiengmay B, Phommasack B, Rim HJ. 2013. Hyperendemicity of *Haplorchis taichui* infection among riparian people in Saravane and Champasak province, Lao PDR. *The Korean Journal of Parasitology* **51**:305-311. doi:10.3347/kjp.2013.51.3.305

Chai JY, Yong TS, Eom KS, Min DY, Shin EH, Banouvong V, Insisiengmay B, Insisiengmay S, Phommasack B, Rim HJ. 2010. Prevalence of the intestinal flukes *Haplorchis taichui* and *H. yokogawai* in a mountainous area of Phongsaly Province, Lao PDR. *The Korean Journal of Parasitology* **48**:339-342. doi:10.3347/kjp.2010.48.4.339

Chaiputcha K, Promthet S, Bradshaw P. 2015. Prevalence and Risk Factors for Infection by *Opisthorchis viverrini* in an Urban Area of Mahasarakham Province, Northeast Thailand. *Asian Pacific Journal of Cancer Prevention* **16**:4173-4176. doi:10.7314 / apjcp.2015.16.10.4173

Chaisiri K, Jollivet C, Della Rossa P, Sanguankiat S, Wattanakulpanich D, Lajaunie C, Binot A, Tanita M, Rattanapikul S, Sutdan D, Morand S, Ribas A. 2018. Parasitic infections in relation to practices and knowledge in a rural village in Northern Thailand with emphasis on fish-borne trematode infection. *Epidemiology & Infection* **147**:1. doi:10.1017/S0950268818002996

Charoensuk L, Subrungruang I, Mungthin M, Pinlaor S, Suwannahitatorn P. 2019. Comparison of stool examination techniques to detect *Opisthorchis viverrini* in low intensity infection. *Acta Tropica* **191**:13-16. doi:10.1016/j.actatropica.2018.12.018

Chuangchaiya S, Laoprom N, Idris ZM. 2019. Prevalence and associated risk factors of *Opisthorchis viverrini* infections in rural communities along the Nam Kam River of Northeastern Thailand. *Tropical Biomedicine* **1**:81-93. http://msptm.org/files/Vol36No1/081-093-Chuangchaiya-S.pdf

Chuong NV, Tuan BV. 2011. Infection status of *O. viverrini* in Đak Môn commune, ĐakGlei district, Kon Tum province. *Practice Medicine* **796**:165-168. (in Vietnamese)

Chuong NV, Tuan BV. 1994. Report on parasitic worm infection in Quang Nam-Da Nang provinces. *Malaria Parasit Dis Prevent Bull* **3**:69. (in Vietnamese)

Chuong NV, Tuan BV, Chau LV. 2000. Research on *Opisthorchis viverrini* in the coastal plain of central Vietnam. Summary record of scientific research works 1996-2000. *National Institute of Malariology, Parasitology and Entomology, Vietnam* **1**:628-635. (in Vietnamese)

Chuong NV, Tuan BV, Chau LV. 1997. Several epidemiological characteristics of *Opisthorchis viverrini*. *Malaria Parasit Dis Prevent Bull* **2**:85-90. (in Vietnamese)

Chuong NV, Tuan BV, Quang HH. 2014. The situation and risk factors of liver fluke infection *Opisthorchis viverrini* in human at two communes in Quang Tri province. *TP. Ho Chi Minh Medicine* **18**:525-532. (in Vietnamese)

Dang TC, Yajima A, Nguyen VK, Montresor A. 2008. Prevalence, intensity and risk factors for clonorchiasis and possible use of questionnaires to detect individuals at risk in northern Vietnam. *Transactions of the Royal Society of Tropical Medicine and Hygiene* **102**:1263-1268. doi:10.1016/j.trstmh.2008.06.002

Dao TT, Bui TV, Abatih EN, Gabriel S, Nguyen TT, Huynh QH, Nguyen CV, Dorny P. 2016. *Opisthorchis viverrini* infections and associated risk factors in a lowland area of Binh Dinh Province, Central Vietnam. *Acta Tropica* **157**:151-157. doi:10.1016/j.actatropica.2016.01.029

De NV, Dat DT, Chau LV. 1997. Investigation results of intestinal helminth infection in the community of several places along the Srepok River (1996-1997). *Pract Health Magazine* **10**:4-8. (in Vietnamese)

De NV, Lam KT, Chau LV. 1996. Liver fluke infection and changes of its infection rates after specific treatment. Summary Record of Scientific Research Works 1991-1996. National Institute of Malariology. *Parasitology and Entomology* **2**:69-77. (in Vietnamese)

De NV, Lam KT, Chau LV. 1998. Study on fluke and tapeworm diseases. *Malaria Parasit Dis Prevent Bull* **2**:29-33. (in Vietnamese)

De NV, Le TH. 2011. Human infections of fish-borne trematodes in Vietnam: prevalence and molecular specific identification at an endemic commune in Nam Dinh province. *Experimental Parasitology* **129**:355-361. doi:10.1016/j.exppara.2011.09.005

Doanh PN, Nawa Y. 2016. *Clonorchis sinensis* and *Opisthorchis spp.* in Vietnam: current status and prospects. *Transactions of the Royal Society of Tropical Medicine and Hygiene* **110**:13-20. doi:10.1093/trstmh/trv103

Doi R, Itoh M, Chakhatrakan S, Uga S. 2017. Epidemiological Investigation of Parasitic Infection of Schoolchildren from Six Elementary Schools in Sakon Nakhon Province, Thailand. *Kobe Journal of Medical Sciences* **62**:E120-E128. JaLCDOI:10.24546/81009703

Duenngai K, Sithithaworn P, Rudrappa UK, Iddya K, Laha T, Stensvold CR, Strandgaard H, Johansen MV. 2008. Improvement of PCR for detection of *Opisthorchis viverrini* DNA in human stool samples. *Journal of Clinical Microbiology* **46**:366-368. doi:10.1128/JCM.01323-07

Echaubard P, Sripa B, Mallory FF, Wilcox BA. 2016. The role of evolutionary biology in research and control of liver flukes in Southeast Asia. *Infection, Genetics and Evolution* **43**:381-397. doi:10.1016/j.meegid.2016.05.019

Eom KS, Yong TS, Sohn WM, Chai JY, Min DY, Rim HJ, Jeon HK, Banouvong V, Insisiengmay B, Phommasack B. 2014. Prevalence of helminthic infections among inhabitants of Lao PDR. *The Korean journal of parasitology* **52**:51-56. doi:10.3347/kjp.2014.52.1.51

Erlanger TE, Sayasone S, Krieger GR, Kaul S, Sananikhom P, Tanner M, Odermatt P, Utzinger J. 2008. Baseline health situation of communities affected by the Nam Theun 2 hydroelectric project in central Lao PDR and indicators for monitoring. *International Journal of Environmental Health Research* **18**:223-242. doi:10.1080/09603120701757815

Feldmeier H, Hazay M, Sato M, Tiengkham P, Nishimoto F, Jiang H, Sopraseuth V, Moji K. 2016. Morbidity assessment of *Opisthorchis viverrini* infection in rural Laos: I. Parasitological, clinical, ultrasonographical and biochemical findings. *Tropical Medicine and Health* **44**:12. doi:10.1186/s41182-016-0012-y

Forrer A, Sayasone S, Vounatsou P, Vonghachack Y, Bouakhasith D, Vogt S, Glaser R, Utzinger J, Akkhavong K, Odermatt P. 2012. Spatial distribution of, and risk factors for, *Opisthorchis viverrini* infection in southern Lao PDR. *PLOS Neglected Tropical Diseases* **6**:e1481. doi:10.1371/journal.pntd.0001481

Giboda M, Ditrich O, Scholz T, Viengsay T, Bouaphanh S. 1991. Current status of food-borne parasitic zoonoses in Laos. *The Southeast Asian journal of tropical medicine and public health* **22 Suppl**:56.

Haswell-Elkins MR, Elkins DB, Sithithaworn P, Treesarawat P, Kaewkes S. 1991. Distribution patterns of *Opisthorchis viverrini* within a human community. *Parasitology* **103**:97-101. doi:10.1017/S0031182000059333

Haswell-Elkins MR, Mairiang E, Mairiang P, Chaiyakum J, Chamadol N, Loapaiboon V, Sithithaworn P, Elkins DB. 1994. Cross-sectional study of *Opisthorchis viverrini* infection and cholangiocarcinoma in communities within a high-risk area in northeast Thailand. *International Journal of Cancer* **59**:505. doi:10.1002/ijc.2910590412

Htun N, Odermatt P, Paboriboune P, Sayasone S, Vongsakid M, Phimolsarn-Nusith V, Tran XD, Ounnavong PS, Andriama-Hefasoa N, Senvanpan ND, Homsana A, Lianosay B, Xayavong D, Robinson DR, Bounsavath P, Prasayasith PP, Syphan SD, Lu YX, Thilakoun K, Xaiyaphet XS, Vongngakesone PT, Eze IC, Imboden M, Sripa B, Reinharz D, Probst-Hensch N. 2018. Association between helminth infections and diabetes mellitus in adults from the Lao People's Democratic Republic: a cross-sectional study. *Infectious Diseases of Poverty* **7**:105. doi:10.1186/s40249-018-0488-2

Hung NM, Dung DT, Lan Anh NT, Van PT, Thanh BN, Van Ha N, Van Hien H, Canh LX. 2015. Current status of fish-borne zoonotic trematode infections in Gia Vien district, Ninh Binh province, Vietnam. *Parasites & Vectors* **8**:21. doi:10.1186/s13071-015-0643-6

Jadsri S, Noojoy A. 1999. A study of liver fluke infection in Sukhothai, Thailand. *The Southeast Asian Journal of Tropical Medicine and Public Health* **30**:588-593. PMID: 10774677

Joob B, Wiwanitkit V. 2017. Prevalence of opisthorchiasis detected by stool examination: Relationship to Chi River system in Thailand. *Annals of Tropical Medicine and Public Health* **10**:293. doi:10.4103/1755-6783.205531

Kaewpitoon N, Kaewpitoon SJ, Meererksom T, Chan-Aran S, Sangwalee W, Kujapun J, Norkaew J, Chuatanam J, Ponpimai S, Pothipim M, Padchasuwan N, Tongtawee T, Matrakool L, Panpimanmas S, Loyd RA, Wakkhuwatthapong P. 2018. Detection of Risk Groups for Carcinogenic Liver Fluke Infection by Verbal Screening Questionnaire Using a Mobile Application. *Asian Pacific Journal of Cancer Prevention* **19**:2013-2019. doi:10.22034/APJCP.2018.19.7.2013

Kaewpitoon SJ, Kaewpitoon N, Rujirakul R, Ueng-Arporn N, Matrakool L, Tongtawee T. 2015. The Carcinogenic Liver Fluke *Opisthorchis viverrini* among Rural Community People in Northeast Thailand: a Cross- Sectional Descriptive Study using Multistage Sampling Technique. *Asian Pacific Journal of Cancer Prevention* **16**:7803-7807. doi:10.7314/apjcp.2015.16.17.7803

Kaewpitoon SJ, Kaewpitoon N, Rujirakul R, Wakkuwattapong P, Matrakul L, Tongtawee T, Loyd RA, Norkaew J, Kujapun J, Chavengkun W, Ponphimai S, Polsripradist P, Eksanti T, Phatisena T. 2016. Nurses and Television as Sources of Information Effecting Behavioral Improvement Regarding Liver Flukes in Nakhon Ratchasima Province, Thailand. *Asian Pacific Journal of Cancer Prevention* **17**:1097-1102. doi:10.7314/apjcp.2016.17.3.1097

Kaewpitoon SJ, Loyd RA, Rujirakul R, Wakkuwattapong P, Tongtawee T, Matrakool L, Panpimanmas S, Kompor P, Norkaew J, Kujapun J, Chavengkun W, Ponphimai S, Pothipim M, Phatisena T, Eksanti T, Polsripradist P, Padchasuwan N, Benjaoran F, Namvichaisirikul N, Kuebkuntod P, Kaewpitoon N. 2016. Primary Care Intervention to Prevent and Control Cholangiocarcinoma: Lesson from Nakhon Ratchasima, Thailand. *Journal of the Medical Association of Thailand* **99 Suppl 7**:S144-S150. PMID: 29901974

Kaewpitoon SJ, Ponphimai S, Pechdee P, Thueng-in K, Khiaowichit J, Meererksom T, Wakhuwatapong P, Bukkhunthod P, Leng M, Namhong T, Taweepakdeechot A, Yardcharoen N, Srithongklang W, Keeratibharat N, Chansangrat J, Kaewpitoon N. 2019. The prevalence of intestinal helminth infection in rural subdistricts of northeastern Thailand. *Tropical Biomedicine* **1**:152-164. http://cmuir.cmu.ac.th/jspui/handle/6653943832/59386

Kaewpitoon SJ, Rujirakul R, Kaewpitoon N. 2012. Prevalence of *Opisthorchis viverrini* infection in Nakhon Ratchasima province, Northeast Thailand. *Asian Pacific Journal of Cancer Prevention* **13**:5245-5249. doi:10.7314/apjcp.2012.13.10.5245

Kaewpitoon SJ, Rujirakul R, Loyd RA, Panpimanmas S, Matrakool L, Tongtawee T, Kompor P, Norkaew J, Chavengkun W, Kujapan J, Polphimai S, Phatisena T, Eaksunti T, Polsripradist P, Padchasuwan N, Kaewpitoon N. 2016. Re-Examination of *Opisthorchis viverrini* in Nakhon Ratchasima Province, Northeastern Thailand, Indicates Continued Needs for Health Intervention. *Asian Pacific Journal of Cancer Prevention* **17**:231-234. doi:10.7314/apjcp.2016.17.1.231

Kaewpitoon SJ, Rujirakul R, Ueng-Arporn N, Matrakool L, Namwichaisiriku N, Churproong S, Wongkaewpothong P, Nimkuntod P, Sripa B, Kaewpitoon N. 2012. Community-based cross-sectional study of carcinogenic human liver fluke in elderly from Surin province, Thailand. *Asian Pacific Journal of Cancer Prevention* **13**:4285-4288. doi: 10.7314/apjcp.2012.13.9.4285

Kaewpitoon SJ, Rujirakul R, Wakkuwattapong P, Matrakool L, Tongtawee T, Panpimanmas S, Kujapun J, Norkaew J, Photipim M, Ponphimai S, Chavengkun W, Kompor P, Padchasuwan N, Sawaspol S, Phandee MC, Phandee W, Phanurak W, Kaewpitoon N. 2016. Overweight Relation to Liver Fluke Infection among Rural Participants from 4 Districts of Nakhon Ratchasima Province, Thailand. *Asian Pacific Journal of Cancer Prevention* **17**:2565-2571. PMID: 27268631

Kaewpitoon SJ, Rujirakul R, Wakkuwattapong R, Matrakool L, Tongtawee T, Panpimanmas S, Pengsaa P, Jomkoa D, Joosiri A, Kaewpitoon N. 2016. *Opisthorchis viverrini* Infection Among People in the Border Areas of Three Provinces, Northeast of Thailand. *Asian Pacific Journal of Cancer Prevention* **17**:2973-2977. PMID: 27356720

Kaewpitoon SJ, Sawaspol S, Phandee MC, Phandee W, Phanurak W, Rujirakul R, Wakkuwattapong P, Matrakool L, Tongtawee T, Panpimanmas S, Benjaoran F, Namvichaisirikul N, Jamkoa D, Joosiri A, Kaewpitoon N. 2016. Analysis of Risk Areas of *Opisthorchis viverrini* in Rural Communities by Using SUT-OV-001. *Journal of the Medical Association of Thailand* **99 Suppl 7**:S138-S143. PMID: 29901970

Kasuya S, Khamboonruang C, Amano K, Murase T, Araki H, Kato Y, Kumada Y, Koyama A, Higuchi M, Nakamura J. 1989. Intestinal parasitic infections among schoolchildren in Chiang Mai, northern Thailand: an analysis of the present situation. *The Journal of Tropical Medicine and Hygiene* **92**:360. PMID: 2810455

Khampitak T, Knowles J, Yongvanit P, Sithithaworn P, Tangrassameeprasert R, Boonsiri P, Satarug S. 2006. Thiamine deficiency and parasitic infection in rural Thai children. *The Southeast Asian Journal of Tropical Medicine and Public Health* **37**:441. PMID: 17120961

Khieu V, Schar F, Marti H, Bless PJ, Char MC, Muth S, Odermatt P. 2014. Prevalence and risk factors of *Strongyloides stercoralis* in Takeo Province, Cambodia. *Parasites & Vectors* **7**:221. doi:10.1186/1756-3305-7-221

Khieu V, Schär F, Marti H, Sayasone S, Duong S, Muth S, Odermatt P. 2013. Diagnosis, treatment and risk factors of *Strongyloides stercoralis* in schoolchildren in Cambodia. *PLOS Neglected Tropical Diseases* **7**:e2035. doi:10.1371/journal.pntd.0002035

Kim JY, Yong TS, Rim HJ, Chai JY, Min DY, Eom KS, Sohn WM, Lim JH, Choi D, Insisiengmay S, Phommasack B, Insisiengmay B. 2018. Ultrasonographic investigation of cholangiocarcinoma in Lao PDR. *Acta Tropica* **182**:128-134. doi:10.1016/j.actatropica.2018.02.031

Kobayashi J, Chinen K, Samidt S, Higa K, Chinen M, Sato Y, Yoshida C. 1999. Prevalence of intestinal parasite infection in toul roka village, phnom penh municipality, Cambodia. *Japanese Journal of Tropical Medicine and Hygiene* **27**:517-519. doi:10.2149/tmh1973.27.517

Kobayashi J, Vannachone B, Sato Y, Manivong K, Nambanya S, Inthakone S. 2000. An epidemiological study on *Opisthorchis viverrini* infection in Lao villages. *The Southeast Asian Journal of Tropical Medicine and Public Health* **31**:128-132. PMID: 11023079

Kobayashi J, Vannachone B, Xeutvongsa A, Manivang K, Ogawa S, Sato Y, Pholsena K. 1996. Prevalence of intestinal parasitic infection among children in two villages in Lao PDR. *The Southeast Asian Journal of Tropical Medicine and Public Health* **27**:562-565. PMID: 9185270

Laoprom N, Laithavewat L, Kopolrat K, Kiatsopit N, Kaewkes S, Chantalux S, Mongkolsin C, Chanmaha B, Andrews RH, Petney TN, Sithithaworn P. 2016. Evaluation of a commercial stool concentrator kit compared to direct smear and formalin-ethyl acetate concentration methods for diagnosis of parasitic infection with special reference to *Opisthorchis viverrini* sensu lato in Thailand. *The Southeast Asian Journal of Tropical Medicine and Public Health* **47**:890-900. PMID: 29620342

Laoraksawong P, Sanpool O, Rodpai R, Thanchomnang T, Kanarkard W, Maleewong W, Kraiklang R, Intapan PM. 2018. Current high prevalences of *Strongyloides stercoralis* and *Opisthorchis viverrini* infections in rural communities in northeast Thailand and associated risk factors. *BMC Public Health* **18**:940. doi:10.1186/s12889-018-5871-1

Laymanivong S, Hangvanthong B, Insisiengmay B, Vanisaveth V, Laxachack P, Jongthawin J, Sanpool O, Thanchomnang T, Sadaow L, Phosuk I, Rodpai R, Maleewong W, Intapan PM. 2016. First molecular identification and report of genetic diversity of *Strongyloides stercoralis*, a current major soil-transmitted helminth in humans from Lao People's Democratic Republic. *Parasitology Research* **115**:2973-2980. doi:10.1007/s00436-016-5052-z

Laymanivong S, Hangvanthong B, Keokhamphavanh B, Phommasansak M, Phinmaland B, Sanpool O, Maleewong W, Intapan PM. 2014. Current status of human hookworm infections, *ascariasis*, *trichuriasis*, *schistosomiasis mekongi* and other trematodiases in Lao People's Democratic Republic. *The American Journal of Tropical Medicine* *and Hygiene* **90**:667-669. doi:10.4269/ajtmh.13-0636

Lee KJ, Bae YT, Kim DH, Deung YK, Ryang YS, Kim HJ, Im KI, Yong TS. 2002. Status of intestinal parasites infection among primary school children in Kampongcham, Cambodia. *The Korean journal of parasitology* **40**:153-155. doi:10.3347/kjp.2002.40.3.153

León TM, Porco TC, Kim CS, Kaewkes S, Kaewkes W, Sripa B, Spear RC. 2018. Modeling liver fluke transmission in northeast Thailand: Impacts of development, hydrology, and control. *Acta Tropica* **188**:101-107. doi:10.1016/j.actatropica.2018.08.008

Lovis L, Mak TK, Phongluxa K, Soukhathammavong P, Sayasone S, Akkhavong K, Odermatt P, Keiser J, Felger I. 2009. PCR Diagnosis of *Opisthorchis viverrini* and *Haplorchis taichui* Infections in a Lao Community in an area of endemicity and comparison of diagnostic methods for parasitological field surveys. *Journal of Clinical Microbiology* **47**:1517-1523. doi:10.1128/JCM.02011-08

Mairiang E, Laha T, Bethony JM, Thinkhamrop B, Kaewkes S, Sithithaworn P, Tesana S, Loukas A, Brindley PJ, Sripa B. 2012. Ultrasonography assessment of hepatobiliary abnormalities in 3359 subjects with *Opisthorchis viverrini* infection in endemic areas of Thailand. *Parasitology International* **61**:208-211. doi:10.1016/j.parint.2011.07.009

Maleewong W, Intapan P, Wongwajana S, Sitthithaworn P, Pipitgool V, Wongkham C, Daenseegaew W. 1992. Prevalence and intensity of *Opisthorchis viverrini* in rural community near the Mekong River on the Thai-Laos border in northeast Thailand. *Journal of the Medical Association of Thailand* **75**:231. PMID: 1402447

Miyamoto K, Kirinoki M, Matsuda H, Hayashi N, Chigusa Y, Sinuon M, Chuor CM, Kitikoon V. 2014. Field survey focused on *Opisthorchis viverrini* infection in five provinces of Cambodia. *Parasitology International* **63**:366-373. doi:10.1016/j.parint.2013.12.003

Nakamura S. 2017. Present Situation of Opisthorchiasis in Vientiane Capital, Lao Peoples' Democratic Republic. *Nihon eiseigaku zasshi. Japanese journal of hygiene* **72**:101-105. doi:10.1265/jjh.72.101

Niamnuy N, Kaewthamasorn M, Congpuong K, Phaytanavanh B, Lohsoonthorn V. 2016. Prevalence and associated risk factors of intestinal parasites in humans and domestic animals across borders of Thailand and Lao PDR: focus on hookworm and threadworm. *The Southeast Asian Journal of Tropical Medicine and Public Health* **47**:901-911. PMID: 29620343

Nithikathkul C, Pumidonming W, Wannapinyosheep S, Tesana S, Chaiprapathong S, Wongsawad C. 2009. *Opisthorchis viverrini* infection in minute intestinal fluke endemic areas of Chiang Mai Province, Thailand. *Asian Biomedicine* **3**:187-191. http://cmuir.cmu.ac.th/jspui/handle/6653943832/59386

Nontasut P, Muennoo C, Sa-nguankiat S, Fongsri S, Vichit A. 2005. Prevalence of strongyloides in Northern Thailand and treatment with ivermectin vs albendazole. *The Southeast Asian Journal of Tropical Medicine and Public Health* **36**:442-444. PMID: 15916052

Ong X, Wang YC, Sithithaworn P, Namsanor J, Taylor D, Laithavewat L. 2016. Uncovering the Pathogenic Landscape of Helminth (*Opisthorchis viverrini*) Infections: A Cross-Sectional Study on Contributions of Physical and Social Environment and Healthcare Interventions. *PLOS Neglected Tropical Diseases* **10**:e5175. doi:10.1371/journal.pntd.0005175

Phimpraphai W, Tangkawattana S, Sereerak P, Kasemsuwan S, Sripa B. 2017. Social network analysis of food sharing among households in opisthorchiasis endemic villages of Lawa Lake, Thailand. *Acta Tropica* **169**:150-156. doi:10.1016/j.actatropica.2017.02.001

Phongluxa K, Xayaseng V, Vonghachack Y, Akkhavong K, van Eeuwijk P, Odermatt P. 2013. Helminth infection in southern Laos: high prevalence and low awareness. *Parasites & Vectors* **6**:328. doi:10.1186/1756-3305-6-328

Piangjai S, Sukontason K, Sukontason KL. 2003. Intestinal parasitic infections in hill-tribe schoolchildren in Chiang Mai, northern Thailand. *The Southeast Asian Journal of Tropical Medicine and Public Health* **34 Suppl 2**:90-93. PMID: 19230577

Prakobwong S, Gunnula W, Chaipibool S, Nimala B, Sangthopo J, Sirivetthumrong N, Ribas A. 2017. Epidemiology of *Opisthorchis viverrini* in an endemic area of Thailand, an integrative approach. *Helminthologia* **54**:298-306. doi:10.1515/helm-2017-0036

Prakobwong S, Suwannatrai A, Sancomerang A, Chaipibool S, Siriwechtumrong N. 2017. A Large Scale Study of the Epidemiology and Risk Factors for the Carcinogenic Liver Fluke *Opisthorchis viverrini* in Udon Thani Province, Thailand. *Asian Pacific Journal of Cancer Prevention* **18**:2853-2860. doi:10.22034/APJCP.2017.18.10.2853

Pumidonming W, Katahira H, Igarashi M, Salman D, Abdelbaset AE, Sangkaeo K. 2018. Potential risk of a liver fluke *Opisthorchis viverrini* infection brought by immigrants from prevalent areas: A case study in the lower Northern Thailand. *Acta Tropica* **178**:213-218. doi:10.1016/j.actatropica.2017.11.023

Pungpak S, Chalermrut K, Harinasuta T, Viravan C, Schelp PF, Hempfling A, Schlattmann P, Bunnag D. 1994. *Opisthorchis viverrini* infection in Thailand: symptoms and signs of infection--a population-based study. *Transactions of the Royal Society of Tropical Medicine and Hygiene* **88**:561-564. doi:10.1016/0035-9203(94)90164-3

Pungpak S, Sornmani S, Suntharasamai P, Vivatanasesth P. 1989. Ultrasonographic study of the biliary system in opisthorchiasis patients after treatment with praziquantel. *The Southeast Asian Journal of Tropical Medicine and Public Health* **20**:157. PMID: 2672363

Radomyos B, Wongsaroj T, Wilairatana P, Radomyos P, Praevanich R, Meesomboon V, Jongsuksuntikul P. 1998. Opisthorchiasis and intestinal fluke infections in northern Thailand. *The Southeast Asian Journal of Tropical Medicine and Public Health* **29**:123-127. PMID: 9740284

Rangsin R, Mungthin M, Taamasri P, Mongklon S, Aimpun P, Naaglor T, Leelayoova S. 2009. Incidence and risk factors of *Opisthorchis viverrini* infections in a rural community in Thailand. The American Journal of Tropical Medicine and Hygiene **81**:152-155. PMID: 19556581

Ribas A, Jollivet C, Morand S, Thongmalayvong B, Somphavong S, Siew C, Ting P, Suputtamongkol S, Saensombath V, Sanguankiat S, Tan B, Paboriboune P, Akkhavong K, Chaisiri K. 2017. Intestinal Parasitic Infections and Environmental Water Contamination in a Rural Village of Northern Lao PDR. *The Korean Journal of Parasitology* **55**:523-532. doi:10.3347/kjp.2017.55.5.523

Rim HJ, Chai JY, Min DY, Cho SY, Eom KS, Hong SJ, Sohn WM, Yong TS, Deodato G, Standgaard H, Phommasack B, Yun CH, Hoang EH. 2003. Prevalence of intestinal parasite infections on a national scale among primary schoolchildren in Laos. *Parasitology Research* **91**:267-272. doi:10.1007/s00436-003-0963-x

Ruankham W, Bunchu N, Koychusakun P. 2014. Prevalence of helminthic infections and risk factors in villagers of Nanglae Sub-District, Chiang Rai Province, Thailand. *Journal of the Medical Association of Thailand* **97 Suppl 4**:S29-S35. PMID: 24851562

Rujirakul R, Ueng-arporn N, Kaewpitoon SJ, Loyd RA, Kaewthani S, Kaewpitoon N. 2015. Risk Areas of Liver Flukes in Surin Province of Thailand using Geographic Information System. *Journal of the Medical Association of Thailand* **98 Suppl 4**:S22-S26. PMID: 26201130

Saengsawang P, Promthet S, Bradshaw P. 2013. Infection with *Opisthorchis viverrini* and use of praziquantel among a working-age population in northeast Thailand. *Asian Pacific Journal of Cancer Prevention* **14**:2963-2966. doi:10.7314/apjcp.2013.14.5.2963

Saengsawang P, Promthet S, Bradshaw P. 2012. Prevalence of OV infection in Yasothon Province, Northeast Thailand. *Asian Pacific Journal of Cancer Prevention* **13**:3399-3402. doi:10.7314/apjcp.2012.13.7.3399

Saenna P, Hurst C, Echaubard P, Wilcox BA, Sripa B. 2017. Fish sharing as a risk factor for *Opisthorchis viverrini* infection: evidence from two villages in north-eastern Thailand. *Infectious Diseases of Poverty* **6**:66. doi:10.1186/s40249-017-0281-7

Saiyachak K, Tongsotsang S, Saenrueang T, Moore MA, Promthet S. 2016. Prevalence and Factors Associated with *Opisthorchis viverrini* Infection in Khammouane Province, Lao PDR. *Asian Pacific Journal of Cancer Prevention* **17**:1589-1593. doi:10.7314/apjcp.2016.17.3.1589

Salao K, Spofford EM, Price C, Mairiang E, Suttiprapa S, Wright HL, Sripa B, Edwards SW. 2020. Enhanced neutrophil functions during *Opisthorchis viverrini* infections and correlation with advanced periductal fibrosis. *International Journal for Parasitology* **50**:145-152. doi:https://doi.org/10.1016/j.ijpara.2019.11.007

Saowakontha S, Pipitgool V, Pariyanonda S, Tesana S, Rojsathaporn K, Intarakhao C. 1993. Field trials in the control of *Opisthorchis viverrini* with an integrated programme in endemic areas of northeast Thailand. *Parasitology* **106 ( Pt 3)**:283-288. doi:10.1017/s0031182000075107

Sato M, Pongvongsa T, Sanguankiat S, Yoonuan T, Dekumyoy P, Kalambaheti T, Keomoungkhoun M, Phimmayoi I, Boupha B, Moji K, Waikagul J. 2010. Copro-DNA diagnosis of *Opisthorchis viverrini* and *Haplorchis taichui* infection in an endemic area of Lao PDR. *The Southeast Asian Journal of Tropical Medicine and Public Health* **41**:28-35. PMID: 20578479

Sato M, Pongvongsa T, Sanguankiat S, Yoonuan T, Kobayashi J, Boupha B, Nishimoto F, Moji K, Sato MO, Waikagul J. 2015. Patterns of trematode infections of *Opisthorchis viverrini* (Opisthorchiidae) and *Haplorchis taichui* (Heterophyidae) in human populations from two villages in Savannakhet Province, Lao PDR. *Journal of Helminthology* **89**:439-445. doi:10.1017/S0022149X14000261

Sayasone S, Mak TK, Vanmany M, Rasphone O, Vounatsou P, Utzinger J, Akkhavong K, Odermatt P. 2011. Helminth and intestinal protozoa infections, multiparasitism and risk factors in Champasack province, Lao People's Democratic Republic. *PLOS Neglected Tropical Diseases* **5**:e1037. doi:10.1371/journal.pntd.0001037

Sayasone S, Odermatt P, Phoumindr N, Vongsaravane X, Sensombath V, Phetsouvanh R, Choulamany X, Strobel M. 2007. Epidemiology of *Opisthorchis viverrini* in a rural district of southern Lao PDR. *Transactions of the Royal Society of Tropical Medicine and Hygiene* **101**:40-47. doi:10.1016/j.trstmh.2006.02.018

Sayasone S, Utzinger J, Akkhavong K, Odermatt P. 2015. Multiparasitism and intensity of helminth infections in relation to symptoms and nutritional status among children: a cross-sectional study in southern Lao People's Democratic Republic. *Acta Tropica* **141**:322-331. doi:10.1016/j.actatropica.2014.09.015

Schär F, Inpankaew T, Traub RJ, Khieu V, Dalsgaard A, Chimnoi W, Chhoun C, Sok D, Marti H, Muth S, Odermatt P. 2014. The prevalence and diversity of intestinal parasitic infections in humans and domestic animals in a rural Cambodian village. *Parasitology International* **63**:597-603. doi:https://doi.org/10.1016/j.parint.2014.03.007

Schurer JM, Ramirez V, Kyes P, Tanee T, Patarapadungkit N, Thamsenanupap P, Trufan S, Grant ET, Garland-Lewis G, Kelley S, Nueaitong H, Kyes RC, Rabinowitz P. 2019. Long-Tailed Macaques (Macaca fascicularis) in Urban Landscapes: Gastrointestinal Parasitism and Barriers for Healthy Coexistence in Northeast Thailand. *The American Journal of Tropical Medicine and Hygiene* **100**:357-364. doi:10.4269/ajtmh.18-0241

Sinuon M, Anantaphruti MT, Socheat D. 2003. Intestinal helminthic infections in schoolchildren in Cambodia. *The Southeast Asian Journal of Tropical Medicine and Public Health* **34**:254-258. PMID: 12971545

Sithithaworn P, Andrews RH, Nguyen VD, Wongsaroj T, Sinuon M, Odermatt P, Nawa Y, Liang S, Brindley PJ, Sripa B. 2012. The current status of opisthorchiasis and clonorchiasis in the Mekong Basin. *Parasitology International* **61**:10-16. doi:10.1016/j.parint.2011.08.014

Sithithaworn P, Sithithaworn J, Srisawangwong T, Tesana S, Daenseekaew W, Fujimaki Y, Ando K. 2003. Epidemiology of *Strongyloides stercoralis* in north-east Thailand: application of the agar plate culture technique compared with the enzyme-linked immunosorbent assay. *Transactions of the Royal Society of Tropical Medicine and Hygiene* **97**:398-402. doi:10.1016/S0035-9203(03)90069-1

Sithithaworn P, Sukavat K, Vannachone B, Sophonphong K, Ben-Embarek P, Petney T, Andrews R. 2006. Epidemiology of food-borne trematodes and other parasite infections in a fishing community on the Nam Ngum reservoir, Lao PDR. *The Southeast Asian journal of tropical medicine and public health* **37**:1083. PMID: 17333758

Sohn W, Jung B, Hong S, Lee K, Park J, Kim H, Cho S, Htoon TT, Tin HH, Chai J. 2019. Low-Grade Endemicity of Opisthorchiasis, Yangon, Myanmar. *Emerging Infectious Diseases* **25**:1435-1437. doi:10.3201/eid2507.190495

Sohn WM, Kim HJ, Yong TS, Eom KS, Jeong HG, Kim JK, Kang AR, Kim MR, Park JM, Ji SH, Sinuon M, Socheat D, Chai JY. 2011. *Echinostoma ilocanum* infection in Oddar Meanchey Province, Cambodia. *The Korean journal of parasitology* **49**:187-190. doi:10.3347/kjp.2011.49.2.187

Sohn WM, Shin EH, Yong TS, Eom KS, Jeong HG, Sinuon M, Socheat D, Chai JY. 2011. Adult *Opisthorchis viverrini* flukes in humans, Takeo, Cambodia. *Emerging Infectious Diseases* **17**:1302-1304. doi:10.3201/eid1707.102071

Sohn WM, Yong TS, Eom KS, Min DY, Lee D, Jung BK, Banouvong V, Insisiengmay B, Phommasack B, Rim HJ, Chai JY. 2014. Prevalence of *Haplorchis taichui* among humans and fish in Luang Prabang Province, Lao PDR. *Acta Tropica* **136**:74-80. doi:10.1016/j.actatropica.2014.04.020

Sohn WM, Yong TS, Eom KS, Pyo KH, Lee MY, Lim H, Choe S, Jeong HG, Sinuon M, Socheat D, Chai JY. 2012. Prevalence of *Opisthorchis viverrini* infection in humans and fish in Kratie Province, Cambodia. *Acta Tropica* **124**:215-220. doi:10.1016/j.actatropica.2012.08.011

Songserm N, Promthet S, Wiangnon S, Sithithaworn P. 2012. Prevalence and co-infection of intestinal parasites among thai rural residents at high-risk of developing cholangiocarcinoma: a cross-sectional study in a prospective cohort study. *Asian Pacific Journal of Cancer Prevention* **13**:6175-6179. doi:10.7314/apjcp.2012.13.12.6175

Sornmani S, Schelp FP, Vivatanasesth P, Patihatakorn W, Impand P, Sitabutra P, Worasan P, Preuksaraj S. 1984. A pilot project for controlling *O. viverrini* infection in Nong Wai, Northeast Thailand, by applying praziquantel and other measures. *Arzneimittel-Forschung* **34**:1231. PMID: 6542402

Sornmani S, Schelp FP, Vivatanasesth P, Pongpaew P, Sritabutra P, Supawan V, Vudhivai N, Egormaiphol S, Harinasuta C. 1981. An investigation of the health and nutritional status of the population in the Nam Pong Water Resource Development Project, northeast Thailand. *Annals of tropical medicine and parasitology* **75**:335. doi: 10.1080/00034983.1981.11687448

Sornmani S, Vivatanasesth P, Impand P, Phatihatakorn W, Sitabutra P, Schelp FP. 1984. Infection and re-infection rates of opisthorchiasis in the water resource development area of Nam Pong project, Khon Kaen Province, northeast Thailand. *Annals of tropical medicine and parasitology* **78**:649-656. doi:10.1080/00034983.1984.11811877

Soukhathammavong P, Odermatt P, Sayasone S, Vonghachack Y, Vounatsou P, Hatz C, Akkhavong K, Keiser J. 2011. Efficacy and safety of mefloquine, artesunate, mefloquine-artesunate, tribendimidine, and praziquantel in patients with *Opisthorchis viverrini*: a randomised, exploratory, open-label, phase 2 trial. *The Lancet Infectious Diseases* **11**:110-118. doi:10.1016/S1473-3099(10)70250-4

Sriamporn S, Pisani P, Pipitgool V, Suwanrungruang K, Kamsa-ard S, Parkin DM. 2004. Prevalence of *Opisthorchis viverrini* infection and incidence of cholangiocarcinoma in Khon Kaen, Northeast Thailand. *Tropical Medicine & International Health* **9**:588-594. doi:10.1111/j.1365-3156.2004.01234.x

Sripa B, Tangkawattana S, Laha T, Kaewkes S, Mallory FF, Smith JF, Wilcox BA. 2015. Toward integrated opisthorchiasis control in northeast Thailand: the Lawa project. *Acta Tropica* **141**:361-367. doi:10.1016/j.actatropica.2014.07.017

Sripa B, Tangkawattana S, Sangnikul T. 2017. The Lawa model: A sustainable, integrated opisthorchiasis control program using the EcoHealth approach in the Lawa Lake region of Thailand. *Parasitology International* **66**:346-354. doi:10.1016/j.parint.2016.11.013

Srivatanakul P, Ohshima H, Khlat M, Parkin M, Sukaryodhin S, Brouet I, Bartsch H. 1991. *Opisthorchis viverrini* infestation and endogenous nitrosamines as risk factors for cholangiocarcinoma in Thailand. *International Journal of Cancer* **48**:821-825. doi:10.1002/ijc.2910480606

Srivatanakul P, Parkin DM, Jiang YZ, Khlat M, Kao-Ian UT, Sontipong S, Wild C. 1991. The role of infection by *Opisthorchis viverrini*, *hepatitis B virus*, and aflatoxin exposure in the etiology of liver cancer in Thailand. A correlation study. *Cancer* **68**:2411-2417. doi:10.1002/1097-0142(19911201)68:11<2411::aid-cncr2820681114>3.0.co;2-0

Strandgaard H, Johansen MV, Aagaard-Hansen J, Petlueng P, Ornbjerg N. 2008. Local perceptions and practices in regard to opisthorchiasis in two villages in Lao PDR. *The Southeast Asian Journal of Tropical Medicine and Public Health* **39**:19-26. PMID: 18567439

Sukontason KL, Sukontason K, Piangjai S, Pungpak S, Radomyos P. 2001. Prevalence of *Opisthorchis viverrini* infection among villagers harboring Opisthorchis-like eggs. *The Southeast Asian Journal of Tropical Medicine and Public Health* **32 Suppl 2**:23-26. PMID: 12041596

Sungkasubun P, Siripongsakun S, Akkarachinorate K, Vidhyarkorn S, Worakitsitisatorn A, Sricharunrat T, Singharuksa S, Chanwat R, Bunchaliew C, Charoenphattharaphesat S, Molek R, Yimyaem M, Sornsamdang G, Soonklang K, Wittayasak K, Auewarakul CU, Mahidol C. 2016. Ultrasound screening for cholangiocarcinoma could detect premalignant lesions and early-stage diseases with survival benefits: a population-based prospective study of 4,225 subjects in an endemic area. *BMC Cancer* **16**:346. doi:10.1186/s12885-016-2390-2

Suwannahitatorn P, Klomjit S, Naaglor T, Taamasri P, Rangsin R, Leelayoova S, Mungthin M. 2013. A follow-up study of *Opisthorchis viverrini* infection after the implementation of control program in a rural community, central Thailand. *Parasites & Vectors* **6**:188. doi:10.1186/1756-3305-6-188

Suwannatrai A, Saichua P, Haswell M. 2018. Epidemiology of *Opisthorchis viverrini* Infection. *Advances in Parasitology* **101**:41. doi: 10.1016/bs.apar.2018.05.002

Tesana S, Srisawangwong T, Sithithaworn P, Itoh M, Phumchaiyothin R. 2007. The ELISA-based detection of anti-*Opisthorchis viverrini* IgG and IgG4 in samples of human urine and serum from an endemic area of north-eastern Thailand. *Annals of Tropical Medicine and Parasitology* **101**:585-591. doi:10.1179/136485907X229068

Thaewnongiew K, Singthong S, Kutchamart S, Tangsawad S, Promthet S, Sailugkum S, Wongba N. 2014. Prevalence and risk factors for *Opisthorchis viverrini* infections in upper Northeast Thailand. *Asian Pacific Journal of Cancer Prevention* **15**:6609-6612. doi: 10.7314/apjcp.2014.15.16.6609

Tomokawa S, Kobayashi T, Pongvongsa T, Nisaygnang B, Kaneda E, Honda S, Moji K, Boupha B. 2012. Risk factors for *Opisthorchis viverrini* infection among schoolchildren in Lao PDR. *The Southeast Asian Journal of Tropical Medicine and Public Health* **43**:574-585. PMID: 23077836

Traub RJ, Macaranas J, Mungthin M, Leelayoova S, Cribb T, Murrell KD, Thompson RC. 2009. A new PCR-based approach indicates the range of *Clonorchis sinensis* now extends to Central Thailand. *PLOS Neglected Tropical Diseases* **3**:e367. doi:10.1371/journal.pntd.0000367

Tungtrongchitr A, Chiworaporn C, Praewanich R, Radomyos P, Boitano JJ. 2007. The potential usefulness of the modified Kato thick smear technique in the detection of intestinal sarcocystosis during field surveys. *The Southeast Asian Journal of Tropical Medicine and Public Health* **38**:232-238. PMID: 17539271

Uengarporn N, Matrakool L, Kaewpitoon SJ, Rujirakul R, Churproong S, Kaewpitoon N. 2016. Nutritional Status among Schoolchildren in the Risk Areas of Liver Fluke Infection, Surin Province, Thailand. *Journal of the Medical Association of Thailand* **99 Suppl 7**:S17-S23. PMID: 29901336

Upatham ES, Viyanant V, Kurathong S, Brockelman WY, Menaruchi A, Saowakontha S, Intarakhao C, Vajrasthira S, Warren KS. 1982. Morbidity in relation to intensity of infection in *Opisthorchiasis viverrini*: study of a community in Khon Kaen, Thailand. *The American Journal of Tropical Medicine and Hygiene* **31**:1156-1163. doi:10.4269/ajtmh.1982.31.1156

Upatham ES, Viyanant V, Kurathong S, Rojborwonwitaya J, Brockelman WY, Ardsungnoen S, Lee P, Vajrasthira S. 1984. Relationship between prevalence and intensity of *Opisthorchis viverrini* infection, and clinical symptoms and signs in a rural community in north-east Thailand. *Bulletin of the World Health Organization* **62**:451-461. PMID: 6331907

Van CD, Doungchawee G, Suttiprapa S, Arimatsu Y, Kaewkes S, Sripa B. 2017. Association between *Opisthorchis viverrini* and *Leptospira spp.* infection in endemic Northeast Thailand. *Parasitology International* **66**:503-509. doi:10.1016/j.parint.2016.10.006

Vannachone B, Kobayashi J, Nambanya S, Manivong K, Inthakone S, Sato Y. 1998. An epidemiological survey on intestinal parasite infection in Khammouane Province, Lao PDR, with special reference to Strongyloides infection. *The Southeast Asian Journal of Tropical Medicine and Public Health* **29**:717-722. PMID: 10772552

Viravan C, Bunnag D, Harinasuta T, Upatham S, Kurathong S, Viyanant V. 1986. Clinical field trial of praziquantel in opisthorchiasis in Nong Rangya Village, Khon Kaen Province, Thailand. *The Southeast Asian journal of tropical medicine and public health* **17**:63. PMID: 3738609

Viyanant V, Brockelman WY, Lee P, Ardsungnoen S, Upatham ES. 1983. A comparison of a modified quick-Kato technique and the Stoll dilution method for field examination for *Opisthorchis viverrini* eggs. *Journal of Helminthology* **57**:191-195. doi:10.1017/S0022149X00009482

Vonghachack Y, Odermatt P, Taisayyavong K, Phounsavath S, Akkhavong K, Sayasone S. 2017. Transmission of *Opisthorchis viverrini*, *Schistosoma mekongi* and soil-transmitted helminthes on the Mekong Islands, Southern Lao PDR. *Infectious Diseases of Poverty* **6**:115-131. doi:10.1186/s40249-017-0343-x

Vonghachack Y, Sayasone S, Bouakhasith D, Taisayavong K, Akkavong K, Odermatt P. 2015. Epidemiology of *Strongyloides stercoralis* on Mekong islands in southern Laos. *Acta Tropica* **141**:289-294. doi:10.1016/j.actatropica.2014.09.016

Vonghachack Y, Sayasone S, Khieu V, Bergquist R, van Dam GJ, Hoekstra PT, Corstjens P, Nickel B, Marti H, Utzinger J, Muth S, Odermatt P. 2017. Comparison of novel and standard diagnostic tools for the detection of *Schistosoma mekongi* infection in Lao People's Democratic Republic and Cambodia. *Infectious Diseases of Poverty* **6**:127. doi:10.1186/s40249-017-0335-x

Waikagul J, Krudsood S, Radomyos P, Radomyos B, Chalemrut K, Jonsuksuntigul P, Kojima S, Looareesuwan S, Thaineau W. 2002. A cross-sectional study of intestinal parasitic infections among schoolchildren in Nan Province, Northern Thailand. *The Southeast Asian journal of tropical medicine and public health* **33**:218. PMID: 12236415

Wang YC, Feng CC, Sithithaworn P. 2013. Environmental determinants of *Opisthorchis viverrini* prevalence in northeast Thailand. *Geospatial Health* **8**:111-123. doi:10.4081/gh.2013.59

Wang YC, Yuen R, Feng CC, Sithithaworn P, Kim IH. 2017. Assessing the role of landscape connectivity on *Opisthorchis viverrini* transmission dynamics. *Parasitology International* **66**:402-412. doi:10.1016/j.parint.2016.06.002

Wangboon C, Worasith C, Thanan R, Eamudomkarn C, Techasen A, Sithithaworn J, Loilome W, Chamadol N, Pinlaor S, Jumnainsong A, Yongvanit P, Khuntikeo N, Bethony JM, Sithithaworn P. 2019. Evaluation of a short term effect of praziquantel treatment in opisthorchiasis-induced hepatobiliary inflammation by urinary 8-oxodG. *Acta Tropica* **189**:124-128. doi:10.1016/j.actatropica.2018.10.003

Waree P, Polseela P, Pannarunothai S, Pipitgool V. 2001. The present situation of paragonimiasis in endemic area in Phitsanulok Province. *The Southeast Asian Journal of Tropical Medicine and Public Health* **32 Suppl 2**:51-54. PMID: 12041605

Warunee N, Choomanee L, Sataporn P, Rapeeporn Y, Nuttapong W, Sompong S, Thongdee S, Bang-On S, Rachada K. 2007. Intestinal parasitic infections among school children in Thailand. *Tropical Biomedicine* **24**:83-88. PMID: 18209713

Watthanakulpanich D, Waikagul J, Maipanich W, Nuamtanong S, Sanguankiat S, Pubampen S, Praevanit R, Mongkhonmu S, Nawa Y. 2010. *Haplorchis taichui* as a possible etiologic agent of irritable bowel syndrome-like symptoms. *The Korean journal of parasitology* **48**:225-229. doi:10.3347/kjp.2010.48.3.225

Watwiengkam N, Sithithaworn J, Duenngai K, Sripa B, Laha T, Johansen MV, Sithithaworn P. 2013. Improved performance and quantitative detection of copro-antigens by a monoclonal antibody based ELISA to diagnose human opisthorchiasis. *Acta Tropica* **128**:659-665. doi:10.1016/j.actatropica.2013.09.012

Wijit A, Morakote N, Klinchid J. 2013. High prevalence of haplorchiasis in Nan and Lampang provinces, Thailand, proven by adult worm recovery from suspected opisthorchiasis cases. *The Korean journal of parasitology* **51**:767-769. doi:10.3347/kjp.2013.51.6.767

Wiwanitkit V, Suwansaksri J, Chaiyakhun Y. 2002. High prevalence of Fasciolopsis buski in an endemic area of liver fluke infection in Thailand. *MedGenMed: Medscape general medicine* **4**:6. PMID: 12466749

Wongba N, Thaewnongiew K, Phathee K, Laithavewat L, Duangsong R, Promthet S, Tangsawad S. 2011. Liver fluke prevention and control in the northeast of Thailand through action research. *Asian Pacific Journal of Cancer Prevention* **12**:1367-1370. PMID: 21875298

Wongsawad C, Phalee A, Noikong W, Chuboon S, Nithikathkul C. 2012. Co-infection with *Opisthorchis viverrini* and *Haplorchis taichui* detected by human fecal examination in Chomtong district, Chiang Mai Province, Thailand. *Parasitology International* **61**:56-59. doi:10.1016/j.parint.2011.10.003

Worasith C, Kamamia C, Yakovleva A, Duenngai K, Wangboon C, Sithithaworn J, Watwiengkam N, Namwat N, Techasen A, Loilome W, Yongvanit P, Loukas A, Sithithaworn P, Bethony JM. 2015. Advances in the Diagnosis of Human Opisthorchiasis: Development of *Opisthorchis viverrini* Antigen Detection in Urine. *PLOS Neglected Tropical Diseases* **9**:e4157. doi:10.1371/journal.pntd.0004157

Worasith C, Wangboon C, Duenngai K, Kiatsopit N, Kopolrat K, Techasen A, Sithithaworn J, Khuntikeo N, Loilome W, Namwat N, Yongvanit P, Carlton EJ, Sithithaworn P. 2019. Comparing the performance of urine and copro-antigen detection in evaluating *Opisthorchis viverrini* infection in communities with different transmission levels in Northeast Thailand. *PLOS Neglected Tropical Diseases* **13**:e7186. doi:10.1371/journal.pntd.0007186

Yajima A, Cong DT, Trung DD, Cam TD, Montresor A. 2009. Cost comparison of rapid questionnaire screening for individuals at risk of clonorchiasis in low- and high-prevalence communities in northern Vietnam. *Transactions of the Royal Society of Tropical Medicine and Hygiene* **103**:447-451. doi:10.1016/j.trstmh.2009.01.007

Yeoh KW, Promthet S, Sithithaworn P, Kamsa-Ard S, Parkin DM. 2015. Re-examination of *Opisthorchis viverrini* Infection in Northeast Thailand. *Asian Pacific Journal of Cancer Prevention* **16**:3413-3418. doi:10.7314/apjcp.2015.16.8.3413

Yong TS, Chai JY, Sohn WM, Eom KS, Jeoung HG, Hoang EH, Yoon CH, Jung BK, Lee SH, Sinuon M, Socheat D. 2014. Prevalence of intestinal helminths among inhabitants of Cambodia (2006-2011). *The Korean journal of parasitology* **52**:661-666. doi:10.3347/kjp.2014.52.6.661

Yong TS, Shin EH, Chai JY, Sohn WM, Eom KS, Lee DM, Park K, Jeoung HG, Hoang EH, Lee YH, Woo HJ, Lee JH, Kang SI, Cha JK, Lee KH, Yoon CH, Sinuon M, Socheat D. 2012. High prevalence of *Opisthorchis viverrini* infection in a riparian population in Takeo Province, Cambodia. *The Korean journal of parasitology* **50**:173-176. doi:10.3347/kjp.2012.50.2.173

Yoon HJ, Ki M, Eom K, Yong T, Chai J, Min D, Rim H, Sohn W, Insisiengmay B, Phommasack B. 2014. Risk factors for *Opisthorchis viverrini* and minute intestinal fluke infections in Lao PDR, 2009-2011. *The American journal of tropical medicine and hygiene* **91**:384-388. doi:10.4269/ajtmh.13-0596

Yoshida I, Horie O, Akkhavong K. 2019. Predictors of hookworm and *Opisthorchis viverrini* infection among adolescents in urban Laos: a cross-sectional study. *Research and Reports in Tropical Medicine* **10**:31-41. doi:10.2147/RRTM.S199577
