# Supplementary material for: Model-based spatial-temporal mapping of opisthorchiasis in endemic countries of Southeast Asia
Source: eLife. 2021 Jan 12;10:e59755. doi: 10.7554/eLife.59755 (PMC7870142; doi:10.7554/eLife.59755)
Supplement: Figure 3—figure supplement 1—source data 1. [file elife-59755-fig3-figsupp1-data1.docx]

**Sensitivity analysis for surveys reported prevalence in intervals**

There was one literature from Suwannatrai and colleagues (***Suwannatrai, et al.***,***2018***) reporting observed prevalence of *O. viverrini* infection in intervals for 77 areas across Thailand. To make full use of all eligible data, we used the median values of the intervals as observed prevalence for our final model. Sensitivity analysis was done by using the lower and the upper limits of the intervals in the modeling analysis and results were compared.

### Figure 3-figure supplement 1-source data 1: Posterior summaries (median and 95% Bayesian credible intervals) of the geostatistical model parameters under different values assigned to prevalence for surveys only reported prevalence in intervals

|  | |  | **Values assigned** |  |
| --- | --- | --- | --- | --- |
|  | | **Median (final model)** | **Lower limit** | **Upper limit** |
| Intercept | | -4.51 (-5.08, -3.94) | -4.61 (-5.18, -4.04) | -4.49 (-5.06, -3.92) |
| Survey type | |  |  |  |
|  | School-based survey | Ref | Ref | Ref |
|  | Community-based survey | 0.96 (0.70, 1.23) | 0.98 (0.71, 1.25) | 0.96 (0.70, 1.23) |
| Diagnostic methods | |  |  |  |
|  | Kato-Katz | Ref | Ref | Ref |
|  | FECT | -0.28 (-0.49, -0.07) | -0.11 (-0.32, 0.09) | -0.30 (-0.51, -0.09) |
|  | Other methods | 0.01 (-0.07, 0.10) | 0.02 (-0.07, 0.10) | 0.01 (-0.07, 0.10) |
| Land surface temperature (LST) in the daytime (℃) | |  |  |  |
|  | < 30.65 | Ref | Ref | Ref |
|  | 30.65 - 32.07 | 0.25 (-0.001, 0.50) | 0.20 (-0.06, 0.44) | 0.25 (0.004, 0.50) |
|  | > 32.07 | 0.07 (-0.18, 0.33) | 0.06 (-0.19, 0.31) | 0.08 (-0.18, 0.33) |
| Human influence index | | -0.01 (-0.02, -0.003) | -0.01 (-0.02, -0.003) | -0.01 (-0.02, -0.003) |
| Distance to the nearest open water bodies | | 0.24 (-1.45, 1.94) | 0.16 (-0.15, 1.86) | 0.24 (-0.46, 1.94) |
| Elevation | | -0.003 (-0.005, -0.001) | -0.003 (-0.006, -0.002) | -0.003 (-0.005, -0.001) |
| Travel time to the nearest big city | | 0.0001 (-0.002, 0.002) | 0.0002 (-0.001, 0.002) | 0.0001 (-0.002, 0.002) |

**Reference**

Suwannatrai A, Saichua P, Haswell M. 2018. Epidemiology of *Opisthorchis viverrini* Infection. *Advances in Parasitology* **101**:41-67. doi:10.1016/bs.apar.2018.05.002
