# Supplementary material for: Model-based spatial-temporal mapping of opisthorchiasis in endemic countries of Southeast Asia
Source: eLife. 2021 Jan 12;10:e59755. doi: 10.7554/eLife.59755 (PMC7870142; doi:10.7554/eLife.59755)
Supplement: Figure 7—source data 1. [file elife-59755-fig7-data1.docx]

**The sources of covariate layers**

There are several kinds of environmental, socioeconomic, and demographic data used in the present study. The sources are given in Figure 7-source data 1.

### Figure 7-source data 1. Covariate layers used in the present study and data sources^a^

| **Data type** | **Source** | **Data period** | **Temporal resolution** | **Spatial resolution** |
| --- | --- | --- | --- | --- |
| NDVI^i^ | MODIS/Terra^b^ | 2000-2018 | 16 days | 1 km |
| LST ^j^ | MODIS/Terra^b^ | 2000-2018 | 8 days | 1 km |
| Land cover | MODIS/Terra^b^ | 2001-2018 | Yearly | 1 km |
| Annual precipitation | WorldClim^c^ | 1960-1990 | - | 1 km |
| Elevation | SRTM^d^ | 2000 | - | 1 km |
| Water bodies | SWBD^e^ | 2000 | - | 30 m |
| Travel time to the nearest big city | MAP^f^ | 2015 | - | 1 km |
| HII^k^ | SEDAC^g^ | 1995-2004 | - | 1 km |
| Population data | SEDAC^g^ | 2015 | - | 5 km |
| Population growth rate^l^ | UN^h^ | - | - | Country-level |

^a^Data asccessed in Janurary 2019

^b^Moderate Resolution Imageing Spectroradiometer (MODIS) /Terra, available at: <https://lpdaac.usgs.gov/>.

^c^Available at: <http://www.worldclim.org/current>/.

^d^Shuttle Radar Topography Mission (SRTM), available at: <https://www2.jpl.nasa.gov/srtm/>.

^e^Shuttle Radar Topography Mission Water Body Data (SWBD), available at: <http://gis.ess.washington.edu/data/vector/worldshore/index.html>/.

^f^D.J. Weiss, A. Nelson, et al. A global map of travel time to cities to assess inequalities in accessibility in 2015. Nature (2018). doi:10.1038/nature25181, available at: <https://map.ox.ac.uk/research-project/accessibility_to_cities/>.

^g^Socioeconomic Data and Applications Center, available at: <http://sedac.ciesin.org/>.

^h^United Nations, available at: <https://population.un.org/wpp/Download/Standard/Population/>.

^i^Normalized difference vegetation index.

^j^Land surface temperature in the daytime and at night.

^k^Human influence index.

^l^This rate was used to project the gridded population of 2015 to 2018 specified by each country with formula $P_{2018}=P_{2015}*e^{T*rate}$.
