# Supplementary material for: Model-based spatial-temporal mapping of opisthorchiasis in endemic countries of Southeast Asia
Source: eLife. 2021 Jan 12;10:e59755. doi: 10.7554/eLife.59755 (PMC7870142; doi:10.7554/eLife.59755)
Supplement: Transparent reporting form [file elife-59755-transrepform.docx]

***eLife’s* transparent reporting form**

We encourage authors to provide detailed information *within their submission* to facilitate the interpretation and replication of experiments. Authors can upload supporting documentation to indicate the use of appropriate reporting guidelines for health-related research (see [EQUATOR Network](http://www.equator-network.org/%20)), life science research (see the [BioSharing Information Resource](https://biosharing.org/)), or the [ARRIVE guidelines](http://www.plosbiology.org/article/info:doi/10.1371/journal.pbio.1000412) for reporting work involving animal research. Where applicable, authors should refer to any relevant reporting standards documents in this form.

If you have any questions, please consult our Journal Policies and/or contact us: [editorial@elifesciences.org](mailto:editorial@elifesciences.org).

**Sample-size estimation**

- You should state whether an appropriate sample size was computed when the study was being designed
- You should state the statistical method of sample size computation and any required assumptions
- If no explicit power analysis was used, you should describe how you decided what sample (replicate) size (number) to use

Please outline where this information can be found within the submission (e.g., sections or figure legends), or explain why this information doesn’t apply to your submission:

This study is an observational study instead of an experimental study. The disease data were collected through a systematic review of literatures following our research protocol with clear inclusion and exclusion criteria (Search strategy, selection criteria, and data extraction, Figure 1-figure supplement 1). We aimed to include all available data that met the criteria (Figure 1). Therefore, sample-size estimation was not applicable. Instead, maps were produced to show the coverages of disease data across the study region in different periods (Figure 2).

**Replicates**

- You should report how often each experiment was performed
- You should include a definition of biological versus technical replication
- The data obtained should be provided and sufficient information should be provided to indicate the number of independent biological and/or technical replicates
- If you encountered any outliers, you should describe how these were handled
- Criteria for exclusion/inclusion of data should be clearly stated
- High-throughput sequence data should be uploaded before submission, with a private link for reviewers provided (these are available from both GEO and ArrayExpress)

Please outline where this information can be found within the submission (e.g., sections or figure legends), or explain why this information doesn’t apply to your submission:

• This study is an observational study instead of an experimental study, thus replicated experiments were not applicable.

• The search process is replicable following the study protocol (Search strategy, selection criteria, and data extraction, Figure 1-figure supplement 1). And the analysis of the data is replicable using the R code provided in this study (available at GitHub when the paper is published: https://github.com/SYSU-Opisthorchiasis).

• Detailed information for each record of disease data can be found in the corresponding original literatures (Figure 2-source data 1, Figure 2-figure supplement 1-source data 1). The sources for data of potential influencing factors were listed in Figure 7-source data 1, where their detailed information can be found.

• The criteria for exclusion/inclusion of data are listed in Materials and Methods (Search strategy, selection criteria, and data extraction).

**Statistical reporting**

- Statistical analysis methods should be described and justified
- Raw data should be presented in figures whenever informative to do so (typically when N per group is less than 10)
- For each experiment, you should identify the statistical tests used, exact values of N, definitions of center, methods of multiple test correction, and dispersion and precision measures (e.g., mean, median, SD, SEM, confidence intervals; and, for the major substantive results, a measure of effect size (e.g., Pearson's r, Cohen's d)
- Report exact p-values wherever possible alongside the summary statistics and 95% confidence intervals. These should be reported for all key questions and not only when the p-value is less than 0.05.

Please outline where this information can be found within the submission (e.g., sections or figure legends), or explain why this information doesn’t apply to your submission:

• Statistical analysis methods are mainly described in Materials and Methods (Model fitting and variable selection, Model validation, sensitivity analysis, and test of preferential sampling).

• The raw data for disease were mapped according to their spatial and temporal distribution in Figure 2, with the corresponding source data (Figure 2-source data 1). The sources of the raw data are listed in Figure 2-source data 1 and Figure 2-figure supplement 1-source data 1.

• As missing information existed in some records, the method for missing data imputation is described in Materials and Methods (Model validation, sensitivity analysis, and test of preferential sampling), and a sensitivity analysis was conducted to evaluate the effects of imputation on missing data, see in Materials and Methods (Model validation, sensitivity analysis, and test of preferential sampling), and Figure 3-figure supplement 1 and its source data.

• As the disease data were collected from different sources, a risk-preferential sampling test was conducted to see whether preferential sampling may exist (Model validation, sensitivity analysis, and test of preferential sampling, Results, Figure 2-source data 2).

• The analysis was under a Bayesian framework. The reported results include the posterior median (Figure 3, Table 2-3, Results) and the estimated uncertainty in the forms of standard error (in Figure 4) and 95% Bayesian Credible interval (Figure 6, Table 2-3, Results). In addition, the posterior probability of the regression coefficient larger than zero was reported in Table 2 for more comprehensive understand whether a factor was a risk or a protective one for the disease.

(For large datasets, or papers with a very large number of statistical tests, you may upload a single table file with tests, Ns, etc., with reference to sections in the manuscript.)

**Group allocation**

- Indicate how samples were allocated into experimental groups (in the case of clinical studies, please specify allocation to treatment method); if randomization was used, please also state if restricted randomization was applied
- Indicate if masking was used during group allocation, data collection and/or data analysis

Please outline where this information can be found within the submission (e.g., sections or figure legends), or explain why this information doesn’t apply to your submission:

Group allocation was not applicable in this study.

**Additional data files (“source data”)**

- We encourage you to upload relevant additional data files, such as numerical data that are represented as a graph in a figure, or as a summary table
- Where provided, these should be in the most useful format, and they can be uploaded as “Source data” files linked to a main figure or table
- Include model definition files including the full list of parameters used
- Include code used for data analysis (e.g., R, MatLab)
- Avoid stating that data files are “available upon request”

Please indicate the figures or tables for which source data files have been provided:

• The source data is provided for all the figures involved in the paper, see Source Data Files.

• The parameters used are described in Materials and Methods (Model fitting and variable selection).

• The R code used for data analysis is uploaded to GitHub (https://github.com/SYSU-Opisthorchiasis), and will be publicly available when the paper is published.
